# Systematic evaluation of long- and short-read RNA-seq for human peripheral blood

**DOI:** 10.1093/narmme/ugag006

**Published:** 2026-01-20

**Authors:** Sadahiro Iwabuchi, Alessandro Nasti, Hikari Okada, Yumie Takeshita, Taka-Aki Sato, Takeshi Urabe, Toshinari Takamura, Takuro Tamura, Atsushi Tajima, Kenichi Matsubara, Shuichi Kaneko

**Affiliations:** Department of Bioinformatics and Genomics, Graduate School of Medical Sciences, Kanazawa University, Kanazawa-shi, Ishikawa 920-8640, Japan; Information-Based Medicine Development, Graduate School of Medical Sciences, Kanazawa University, Kanazawa-shi, Ishikawa 920-8640, Japan; Information-Based Medicine Development, Graduate School of Medical Sciences, Kanazawa University, Kanazawa-shi, Ishikawa 920-8640, Japan; Department of Endocrinology and Metabolism, Graduate School of Medical Sciences, Kanazawa University, Kanazawa-shi, Ishikawa 920-8640, Japan; iLAC Co., Ltd, Tsukuba-shi, Ibaraki 305-8550, Japan; Department of Gastroenterology, Public Central Hospital of Matto, Hakusan-shi, Ishikawa 924-8588, Japan; Department of Endocrinology and Metabolism, Graduate School of Medical Sciences, Kanazawa University, Kanazawa-shi, Ishikawa 920-8640, Japan; Research and Development Center for Precision Medicine, University of Tsukuba, Tsukuba-shi, Ibaraki 305-8550, Japan; Department of Bioinformatics and Genomics, Graduate School of Medical Sciences, Kanazawa University, Kanazawa-shi, Ishikawa 920-8640, Japan; iLAC Co., Ltd, Tsukuba-shi, Ibaraki 305-8550, Japan; Information-Based Medicine Development, Graduate School of Medical Sciences, Kanazawa University, Kanazawa-shi, Ishikawa 920-8640, Japan

## Abstract

RNA sequencing (RNA-seq) technologies enable comprehensive transcriptomic profiling, yet systematic comparisons using identical biological samples remain limited. Here, we performed a multi-faceted comparison of long-read (PacBio) and short-read (Illumina) RNA-seq using the same RNA from peripheral blood cells of four healthy donors. Unlike prior studies that aggregate datasets from different sources, this study evaluates platform-dependent performance across gene expression, transcript variants, fusion genes, primary microRNAs (pri-miRNAs), and immune receptor complementarity-determining region 3 (CDR3) regions using widely available software, highlighting both reproducibility and accessibility. Long-read sequencing outperformed short-read sequencing in detecting complex alternative splicing events, novel transcript isoforms, and full-length immune receptor sequences, particularly immunoglobulin heavy chains, enhancing clonotype resolution. Both platforms captured largely overlapping pri-miRNAs and CDR3 sequences, but each also detected unique elements, demonstrating that total RNA can serve as a proxy for these specialized features when dedicated kits are not used. Short-read sequencing retained superior quantification accuracy for highly expressed genes and stronger concordance with microarray data. Collectively, our findings reveal the complementary strengths of long- and short-read RNA-seq and provide a practical framework for systematic, side-by-side comparison of transcriptomic features, emphasizing the benefits of using the same input material and standard analysis pipelines.

## Graphical Abstract

**Graphical Abstract:**
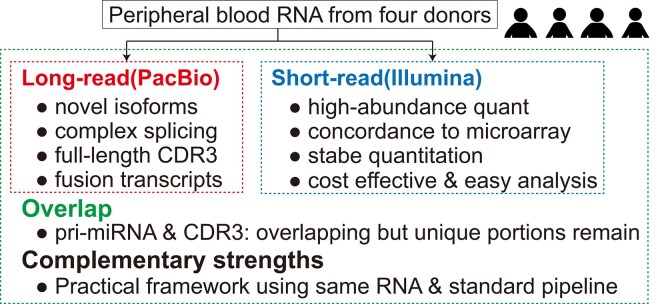

## Introduction

RNA sequencing (RNA-seq) has revolutionized transcriptomic analysis by enabling high-throughput profiling of gene expression, transcript variants, and immune repertoires. While most studies have relied on short-read sequencing due to its high accuracy, cost-effectiveness, and mature analytical pipelines [1], such approaches inherently struggle with reconstructing full-length transcripts, identifying complex splicing events, and detecting long non-coding RNAs (lncRNAs) or fusion transcripts. Recent advances in long-read sequencing, including platforms from Oxford Nanopore Technologies (ONT) and Pacific Biosciences (PacBio), have overcome some of these limitations and enabled more complete characterization of transcriptomes [2]. However, despite these technological advances, few studies have directly and comprehensively compared long- and short-read RNA-seq across multiple transcriptomic dimensions using identical biological samples. Moreover, no prior work to our knowledge has simultaneously examined not only gene expression but also isoform diversity, lncRNAs, fusion transcripts, microRNAs, and immune receptor repertoires that were all derived from matched patient samples. Several recent studies have addressed the capabilities of long-read sequencing in specific biological contexts. For example, Inamo *et al.* systematically profiled 29 sorted immune cell subsets using both long- and short-read RNA-seq to reveal disease-associated isoforms, but did not perform comparisons using the same RNA input across platforms [3]. Similarly, the ENCODE4 project applied long- and short-read RNA-seq to diverse human and mouse tissues to generate transcriptomic annotations, but the samples were not matched between technologies [4]. Recent work benchmarked long-read RNA-seq protocols for sensitivity and quantification accuracy yet focused on method comparison rather than biological sample consistency [5]. These studies underscore the power of long-read sequencing but fall short of providing a unified, within-sample performance evaluation across multiple RNA classes.

ONT employs nanopore-based sequencing that captures electrical current changes as nucleic acid strands pass through protein nanopores [2]. While this enables ultra-long reads that are useful in resolving repetitive or complex genomic regions [6], it is also susceptible to high base-calling error rates due to noisy signal profiles [7]. PacBio’s single-molecule, real-time (SMRT) sequencing, on the other hand, uses zero-mode waveguides (ZMWs) and fluorescently labeled nucleotides to achieve highly accurate long reads through circular consensus sequencing (CCS) [8]. Although PacBio is more expensive and requires specialized bioinformatics tools such as Iso-Seq3 and TAMA [9–11], it provides an optimal balance between read length and base accuracy for full-length transcript identification. Recent work has applied long-read RNA-seq to study diverse transcriptomic features such as circular RNAs [12], neurodevelopmental isoforms [13, 14], and integration with single-cell RNA-seq datasets [15–17]. A major benchmark study (SG-NEx) showed that both ONT and PacBio outperform short-read protocols in isoform and splice-junction detection [2, 18]. However, these studies typically focus on individual transcriptomic features or cell types and do not offer a unified evaluation across multiple RNA species from matched biological samples.

In this study, we present a systematic comparison of long-read (PacBio) and short-read (Illumina) RNA-seq using blood cells obtained from the same individuals. We analyzed gene expression, transcript isoforms, lncRNAs, fusion genes, primary microRNAs (pri-miRNAs), and immune receptor repertoires, including CDR3 sequences. This integrative analysis offers a unique opportunity to benchmark the relative performance of long- and short-read technologies across a wide spectrum of transcriptomic features. Our findings reveal the complementary strengths of both platforms and emphasize the added value of long-read sequencing in capturing biologically relevant transcript diversity.

## Materials and methods

### RNA acquisition and processing

Long-read RNA-seq was performed using blood-derived RNA, and the raw data were shared with a related study [19]. In brief, total RNA was extracted from PAXgene blood RNA samples from four participants (males: *n* = 2; females: *n* = 2), who visited the Public Central Hospital of Matto Ishikawa for medical examinations in 2016, as previously described [20]. High-quality RNA (RIN ≥ 7) was reverse transcribed and amplified using the Iso-Seq Express 2.0 kit (PacBio, Menlo Park, CA, USA). Library quantification showed consistent cDNA input amounts (187–262 ng) and library concentrations (2.9–4.0 nM), and sequencing was performed on the Sequel IIe instrument using the DNA Binding Kit v3.1 with a 2 h pre-extension and a 24 h movie time, following PacBio-recommended conditions. Quality control metrics for long-read libraries and sequencing were obtained from the SMRT Link and laboratory QC reports. All four samples yielded stable HiFi read outputs (8.98–9.56 Gb per sample), with high P1 percentages (88.8%–95.4%), indicating excellent HiFi purity across runs. MultiNA and smear analyses demonstrated broad fragment distributions ranging from 500 to 11 000 bp, with sharp peaks centered ~2 kb for all samples (2163, 2106, 2268, and 2143 bp). These profiles confirm that the Iso-Seq libraries captured full-length transcripts with minimal short-fragment over-representation. The same RNA aliquots were used for both long- and short-read RNA library preparation. Detailed methods for the short-read RNA-seq library preparation, sequencing, and analysis are described below. Library preparation was performed by DNA Chip Research Inc. (Tokyo, Japan) using the NEBNext Ultra II RNA Directional Library Prep Kit for Illumina (New England Biolabs, Ipswich, MA, USA). The libraries were sequenced on a NextSeq platform (Illumina, San Diego, CA, USA) at the Kazusa DNA Research Institute (Chiba, Japan). Short-read RNA-seq was performed using single-end 76-bp reads [19]. Two libraries were sequenced across multiple lanes, and the reads from different lanes were merged for downstream analyses, resulting in a total of 28 043 629 and 25 929 399 reads, respectively. The other two libraries were sequenced on a single lane, yielding 160 496 176 and 162 249 399 reads, respectively. External RNA spike-in controls such as ERCC standards were not included in the library preparation for either long-read or short-read RNA-seq. Because identical RNA aliquots were used for both platforms and downstream analyses relied on internal normalization methods (e.g. DESeq2 size-factor normalization), platform comparisons were performed without external spike-in-based calibration. The study protocol was approved by the review committees of Kanazawa University (Human Genome and Genetic Analysis Committee; protocol number: 2015–007; https://www.med.kanazawa-u.ac.jp/staff/ethics/genome/index.html) and by the Matto Ishikawa Public Central Hospital. The principles of the Declaration of Helsinki were followed during the study, and the observational study was registered with the University Hospital Medical Information Network (UMIN; Clinical Trials Registry, no.: UMIN000051647). In the present study, we utilized long-read RNA-seq data derived from four clinically healthy individuals. These four participants were selected from a cohort of 61 health-check examinees based on the absence of clinical abnormalities [20], and their long-read RNA-seq data had been previously published [19].

### Data analysis and visualization

The obtained raw data (PacBio long-read: BAM and BAI files; Illumina short-read: FASTQ files) were imported into CLC Genomics Workbench (version 25.0.1; QIAGEN, Hilden, Germany) for downstream analysis [21]. In both datasets, sequence quality was first assessed using the “Prepare Sequencing Reads” and “QC for Sequencing Reads2” tools with default setting. Adapter trimming and quality filtering were subsequently performed using the “Trim Reads” tool, which removed platform-specific adapters and low-quality sequences. For short-read RNA-seq, analysis was conducted using the “RNA-Seq Analysis workflow,” which incorporates splice-aware mapping algorithms comparable to STAR, enabling accurate quantification of gene expression and identification of exon–exon junctions. Sequence alignment was performed which employs a splice-aware alignment algorithm functionally comparable to STAR. While CLC’s internal aligner is proprietary and implemented in Java, its performance has been benchmarked to yield gene quantification results consistent with STAR and other standard aligners [22]. For long-read RNA-seq, the “RNA-Seq Analysis for Long Reads” module was applied, optimized for full-length transcript detection and isoform-level resolution, including improved capture of exon connectivity. Sequence reads from both platforms were mapped to the complete human telomere-to-telomere (T2T) reference genome (T2T-CHM13v2.0, GCA_009914755.4, Jan 24, 2022). Mapping statistics, including total reads, mapping rates, gene and exon coverage, exon–exon junction detection, and intergenic read proportions, are summarized in Supplementary Table S1. Notably, long-read RNA sequencing captured a higher proportion of exon-exon junctions compared to short-read data, highlighting its superior capability to identify full-length splice variants and complex transcript isoforms.

Transcriptomic data (TPM values) derived from long- and short-read RNA-seq were analyzed using R (v4.3.2) and RStudio (v2025.05.1 + 524) with default setting. All statistical analyses and data visualization were conducted in R with standard packages, including dplyr, ggplot2, ggrepel, pheatmap, and clusterProfiler. Transcriptomic data (TPM values) derived from long- and short-read RNA-seq were analyzed using R (v4.3.2) and RStudio (v2025.05.1 + 524) with default setting. All statistical analyses and data visualization were conducted in R with standard packages, including dplyr, ggplot2, ggrepel, pheatmap, and clusterProfiler. TPM values were used solely for visualization purposes (e.g. volcano plots, heatmaps, and rank-based analyses), as TPM enables intuitive comparison of expression magnitudes across genes; TPM values were not used for statistical inference. For differential gene expression analysis, raw count data were analyzed using DESeq2 (v1.40.2) with a paired design, treating donor identity as a blocking factor and sequencing platform (long-read versus short-read) as the primary variable. Differentially expressed genes (DEGs) were defined as those with adjusted *P*-value < 0.05 and |log2 fold change| ≥ 1. Long-specific genes, short-specific genes, and common genes were defined based on DESeq2 results (long > short, short > long, or adjusted *P* ≥ 0.05, respectively). Volcano plots were generated using ggplot2 and ggrepel based on TPM-derived fold changes for visualization, with threshold lines set at log2FC = ± 2 and *P*-value = 0.01 [–log10(*P*) = 2]. These thresholds were used only for visual display and not for statistical significance assessment. Residual analysis was also performed to evaluate consistency and dispersion of gene expression differences across samples and plotted to assess outliers or unexplained variance between conditions. MA plots (mean–average plots) were generated to visualize the relationship between the average gene expression level and the magnitude of expression change (log_2_ fold change) between long- and short-read groups. Heatmaps were generated using the pheatmap package based on log10-transformed TPM values (+1 pseudocount). Row-wise *Z*-score normalization was applied to highlight gene-specific variation. Hierarchical clustering was performed using correlation-based distance and the Ward.D2 linkage method. Heatmaps were used to visualize global expression patterns across long- and short-read samples. Gene symbols were converted to Entrez IDs using the bitr function from the clusterProfiler package and the org.Hs.eg.db annotation database. Gene ontology (GO) enrichment analysis was conducted for biological process (BP), cellular component (CC), and molecular function (MF) terms using enrichGO. KEGG pathway enrichment was performed using enrichKEGG (organism = “hsa”, *P*-value < 0.05). All GO and (KEGG) enrichment analyses were performed using gene sets defined by DESeq2. To further benchmark the RNA-seq platforms against an established profiling technology, we incorporated microarray data obtained from the same blood samples [20]. Rank-based analyses were also performed to assess concordance between RNA-seq data and microarray data. Genes were ranked by expression level within each platform, and absolute rank differences between RNA-seq (long-read or short-read) and microarray were computed. Boxplots were used to visualize the dispersion of rank differences, while scatter plots of RNA-seq rank versus microarray rank were used to assess overall concordance and identify systematic biases. Additionally, ranked log fold-change plots were created to display the distribution of gene-wise expression differences [log_10_(Long) – log_10_(Short)] across genes ordered by rank.

### Transcript discovery and classification

Transcript discovery was performed using the QIAGEN CLC Genomics Workbench. The built-in “Transcript Discovery” pipeline (version 25.0.1) was applied to both long- and short-read RNA-seq datasets using default parameters. Reads were aligned to the complete human T2T-CHM13v2.0 reference genome. Default pipeline parameters were used for transcript detection, and details of specific distance-based parameters were applied to classify transcripts. The analysis generated novel and unknown transcript isoforms, including gene assignments, transcription start and end sites, and splicing annotations. Similar workflows have been applied in prior studies to investigate isoform diversity in stem cells and to characterize previously unannotated transcripts in non-model organisms [23]. For each sample, CLC identified transcript structures by assigning genomic coordinates (chromosome, start, and end positions), transcript length, and gene names when available. It also provided counts of total and spliced reads for each transcript. CLC automatically classified unannotated transcripts into two categories: “Unknown transcripts,” which lacked correspondence with any known gene models, and “Novel spliced transcripts,” which represented new splice variants inferred from the sequencing data. For each of these transcript classes, we calculated the total number detected in each sample and summarized the results across groups as mean ± standard error. Among the novel variants, we distinguished two forms: (i) transcripts assigned to known gene names but located at novel genomic positions, and (ii) transcripts labeled as “Gene_X,” which represent de novo predictions without existing gene annotations. Classification into Gene_X was determined based on structural criteria, including gene merging and exon merging distances, which were set at 50 and 100 bp, respectively. For each transcript class, the total number detected in each sample was calculated and summarized across groups as mean ± standard error. It should be noted that optional functional prediction analyses, such as ORF detection or sequence conservation, were not performed by the CLC pipeline and thus were not part of the classification. This purely structural approach provides reproducible and transparent definitions for novel transcript identification, facilitating cross-platform and cross-sample comparisons.

### Fusion transcript detection and comparative analysis using FusionGDB2

Fusion transcript detection was performed using FusionGDB2 (ver. 2024-12), which contains 65 536 reference fusion genes. Raw FASTQ files from both long- and short-read RNA-seq datasets were processed to map reads to the FusionGDB2 reference [24]. Detected fusion transcripts were quantified based on read counts normalized to the total number of mapped reads in each sample, providing semi-quantitative expression estimates. To compare platform-specific detection patterns, the mapping rates and normalized expression levels of fusion transcripts were calculated for each platform. For selected fusion transcripts, visual inspection of read alignments was conducted to confirm full-length coverage across fusion junctions. Correlation analyses between transcript length and normalized read counts were performed to assess potential biases in detection efficiency. This approach enabled systematic comparison of long- and short-read RNA-seq platforms, highlighting differences in fusion transcript capture and the advantages of long-read sequencing for complex fusion events.

### Detection of pri-miRNAs and microRNA network analysis

Because the RNA libraries were generated using poly(A)-selected total RNA, the microRNA (miRNA)-related reads detected in this study predominantly represent polyadenylated pri-miRNA transcripts rather than mature miRNAs. Mature miRNAs were not efficiently captured by poly(A)-based RNA-seq, and therefore we did not attempt to quantify mature miRNA abundance directly. Instead, we treated pri-miRNA detection as a semi-quantitative measure of transcription at miRNA host loci. Furthermore, downstream analyses involving mature miRNAs (e.g. target–gene inference and enrichment analyses) were performed in an annotation-based manner using miRBase and miRNet, which link each pri-miRNA to its corresponding mature miRNAs. Thus, these analyses should be interpreted as exploratory, reflecting potential regulatory landscapes rather than direct evidence of active miRNA-mediated regulation in blood.

The detection of pri-miRNAs and downstream analyses were conducted based on annotations from miRBase v22, which includes 1115 human mature miRNAs and over 38 000 miRNAs from other organisms. For pri-miRNA detection, all raw FASTQ reads from both long- and short-read RNA-seq were directly mapped to the human pri-miRNA reference sequences obtained from miRBase v22, rather than relying on the genome-based T2T mapping. This approach allowed us to capture pri-miRNAs that may not be fully represented in the T2T-CHM13v2.0 genome annotation. In the CLC Genomics Workbench, short-read data can be processed using the built-in “miRNA analysis” workflow optimized for small RNA-seq; however, this workflow is not compatible with long-read sequencing data. Therefore, for both platforms, read files were directly mapped against the human pri-miRNA reference sequences (miRBase v22) using a minimap2-based alignment algorithm embedded in CLC, ensuring consistent analysis across platforms while accommodating the structural characteristics of long-read data [25]. Detected pri-miRNAs were quantified based on read counts relative to the total number of mapped reads, providing a semi-quantitative measure of expression. Detected pri-miRNAs from each sequencing platform were then subjected to network and functional analyses. miRNA–target gene interactions were retrieved from the miRNet database (https://www.mirnet.ca/) by selecting human miRNAs using their miRBase IDs. Experimentally validated target information from miRTarBase v9.0 was integrated along with protein–protein interaction (PPI) data and transcription factor–mature miRNA (TF-miRNA) regulatory relationships. For each platform (long-read and short-read) and for the commonly detected set (common), the top-ranking miRNAs were identified based on the number of target genes. Shared target genes among these top miRNAs were used to construct the corresponding platform-specific miRNA-target gene subnetworks. Network visualization was performed using graph-based layout algorithms: miRNA nodes were colored red (long-read), blue (short-read), or purple (common), while target gene nodes were black. Both labeled and unlabeled versions of the networks were created for interpretability. To further explore the functional architecture of miRNA-regulated genes, PPI networks were constructed based on the target genes of top miRNAs in each group. PPI information was sourced from the STRING database and visualized in R using the igraph and ggraph packages. Community detection was performed using the Louvain algorithm, allowing identification of modular structures and clustering patterns within the gene networks. These analyses enabled a comprehensive comparison of platform-specific miRNA regulatory landscapes and highlighted the advantages of long-read sequencing in capturing polyadenylated pri-miRNAs and their complex target interactions.

### TCR and BCR repertoire analysis using MiXCR

To evaluate the immunoreceptor repertoires from bulk RNA-seq data, all raw FASTQ files from long- and short-read RNA-seq datasets were analyzed using MiXCR (v4.3.2), a widely used tool for profiling T- and B-cell receptor sequences from high-throughput sequencing data [26]. Analyses were performed using default parameters, ensuring that the same pipeline and settings were applied to both platforms. This pipeline conducted alignment, clonotype assembly, and CDR3 annotation. Separate analyses were performed for each of the immunoglobulin chains (IGH, IGK, and IGL) and T-cell receptor (TCR) chains (TRA, TRB, TRD, and TRG). Each resulting clones.tsv file was processed in R using custom scripts. Read counts were normalized by the total number of mapped reads in each sample to account for differences in sequencing depth and minimize bias. Shannon and Simpson diversity indices were then calculated using the vegan package, providing semi-quantitative measures of repertoire diversity. Gene pairing (e.g. IGHV-IGHJ and TRAV-TRAJ) was visualized as heatmaps using ggplot2. Motif analysis of CDR3 sequences was performed with the ggseqlogo package in R, generating sequence logos separately for nucleotide and amino acid sequences. For amino acid motifs, residues were color-coded according to biochemical properties (acidic, basic, hydrophobic, and polar). This approach allowed consistent and comparable characterization of TCR and BCR repertoires across both long- and short-read RNA-seq platforms, accounting for differences in sequencing depth and enabling downstream diversity and motif analyses.

### Statistical analysis

**Figure 1.**
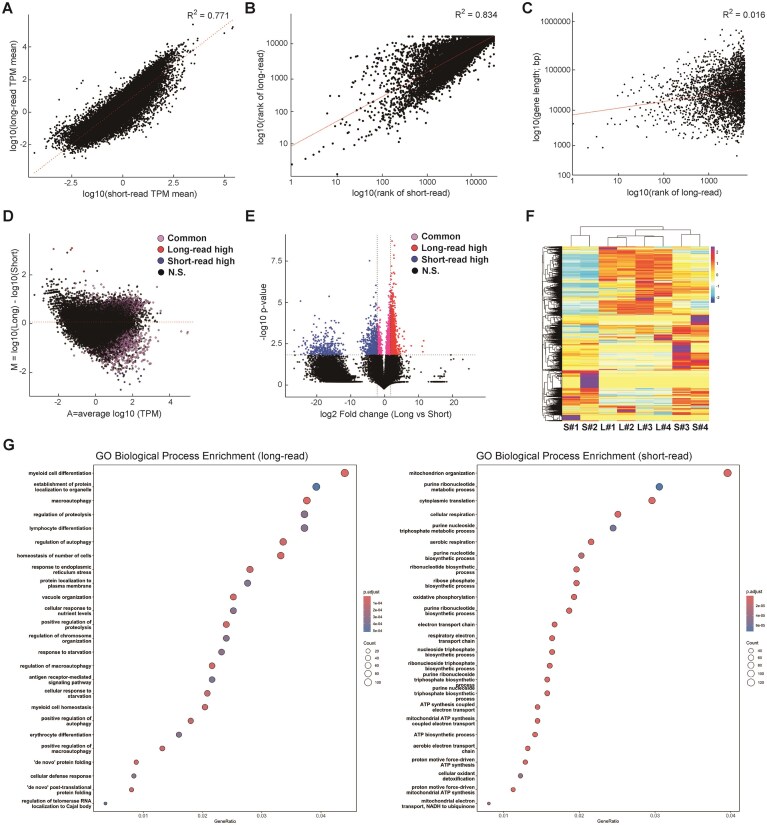
Comparative transcriptome profiling using long- and short-read RNA-seq technologies. (**A**) Scatter plot showing the relationship between average TPM values of genes detected by long- and short-read RNA-seq across all samples. Each point represents a gene. The red dotted line indicates the linear regression fit, with the coefficient of determination (*R*^2^) displayed on the plot. (**B**) Scatter plot showing the correlation between gene expression ranks derived from short-read and long-read RNA-seq data. Genes were ranked based on average TPM values in descending order within each method, and log_10_-transformed ranks were plotted on the *x*-axis (short-read) and *y*-axis (long-read). Each point represents a gene, with a red dotted line indicating the linear regression fit. The *R*^2^ quantifies the similarity in gene detection rankings between the two sequencing platforms. (**C**) Scatter plot illustrating the association between gene expression rank and gene length for the top 1000 genes with higher expression in long-read RNA-seq compared to short-read. The *x*-axis shows the log_10_-transformed rank of genes based on long-read expression, while the *y*-axis displays the log_10_-transformed gene length (in base pairs). A red regression line with the *R*^2^ is included to assess whether gene length influences the detection or quantification of genes with elevated expression in long-read sequencing. (**D**) MA plot illustrating the average log10-transformed TPM (A) against the log_10_ fold change (M) between long- and short-read datasets. Red indicates genes significantly upregulated in long-read data (*P* < 0.01, |log_10_ fold change| ≥ 2); blue indicates genes significantly upregulated in short-read data (*P* < 0.01, |log_10_ fold change| ≥ 2); violet indicates genes with significant *P*-value (*P* < 0.01) but low fold change (|log_10_ fold change| < 2); and black indicates genes not significantly different (*P* ≥ 0.01). The horizontal dashed line indicates zero log fold change. (**E**) Volcano plot displaying log_2_ fold change versus −log_10_ adjusted *P*-value for genes comparing long- and short-read data. Genes significantly upregulated in long-read data (log_2_ fold change > 2, adjusted *P* < 0.01) are shown as red circles; those upregulated in short-read as blue circles; and genes common to both as violet circles (−2 < log_2_ fold change < 2, adjusted *P* < 0.01), all with black borders and white fill. Threshold lines for fold change and *P*-value significance are included as dashed lines. (**F**) Heatmap of log_10_-transformed TPM values for genes across short-read (*n* = 4) and long-read (*n* = 4) RNA-seq samples from each patient. Each row represents a gene, and each column represents an individual sample. TPM values were log-transformed (+1) and row-normalized (*Z*-score). Genes and samples were hierarchically clustered using correlation distance and the Ward.D2 method. The color gradient reflects relative gene expression levels, ranging from blue (low expression) to red/purple (high expression). S#1 to S#4 or L#1 to L#4 indicate short-read or long-read RNA samples from patients #1 to #4, respectively. (**G**) Dot plot showing Gene Ontology (GO) Biological Process enrichment results for genes significantly differentially expressed between long- and short-read data (adjusted *P*-value < 0.05), as determined by DESeq2 paired differential analysis. Terms enriched in long-upregulated and short-upregulated genes are displayed, with dot size indicating gene ratio and color representing adjusted *P*-value.

All analyses were performed using R (v4.3.2) and RStudio (v2025.05.1 + 513) with standard packages including dplyr, ggplot2, ggrepel, and pheatmap. For expression data (TPM values) obtained from long- and short-read RNA-seq, values were log-transformed (log_10_ or log_2_, as appropriate) prior to comparison across platforms. Genome-wide differential expression was reanalyzed using raw count data with DESeq2, which models count-based distributions and applies Benjamini–Hochberg false discovery rate (FDR) correction. Genes with adjusted *P*-values (padj) < 0.05 and |log2 fold change| ≥ 1 were considered significantly differentially expressed. These DESeq2-derived DEGs were used for enrichment analyses (Fig. 1G and Supplementary Fig. S1B). For count-based comparisons, including the number of unknown transcripts, novel spliced transcripts, or spliced-to-total read ratios, data were analyzed without log transformation. Group comparisons were conducted using one-way ANOVA followed by Tukey’s post-hoc test, or Kruskal–Wallis’s rank-sum test when normality could not be assumed. For comparisons between RNA-seq and microarray data, rank-based analyses were performed to assess concordance. Genes were ranked by expression level within each platform, and absolute rank differences between RNA-seq (long-read or short-read) and microarray were calculated. Scatter plots of RNA-seq rank versus microarray rank and boxplots of rank differences were used for visualization. Linear regression models were fitted to log-transformed expression values to estimate correlation coefficients (*R*²), but no statistical significance tests were applied for these comparisons, as these analyses were intended for exploratory visualization of concordance. All read count-based metrics, including TCR/BCR diversity indices and fusion transcript detection counts, were normalized to the total number of mapped reads per sample to account for differences in sequencing depth. Shannon and Simpson diversity indices for TCR and BCR repertoires were calculated using the vegan package in R.

## Results

### Correlation and expression differences between sequencing platforms

To evaluate transcriptomic consistency across sequencing platforms, we compared average TPM values obtained from long- and short-read RNA-seq of blood cells from four healthy individuals. Sequencing depth and exon-exon junction coverage are summarized in Supplementary Table S1. The long-read data yielded ~7.6 million reads in total, whereas the short-read platform generated an average of 138.2 million reads per sample, totaling ~553 million reads, corresponding to ~72-fold higher total read counts. A scatter plot of all genes revealed a strong correlation in expression levels between the two platforms (*R*^2^ = 0.771; Fig. 1A). Ranking genes by average TPM within each platform and plotting these ranks (log_10_-transformed) against each other also showed high concordance (*R*^2^ = 0.834; Fig. 1B), indicating that highly expressed genes were similarly ranked across platforms. To assess whether long-read-specific high-expression genes were biased by gene length, we plotted their expression rank against gene length (log_10_-transformed), revealing a negligible correlation (*R*^2^ = 0.016; Fig. 1C). These results suggest that gene length had minimal impact on platform-specific detection. Expression differences were further visualized using an MA plot (Fig. 1D), a rank plot (Supplementary Fig. S1A), and a volcano plot (Fig. 1E). The number of genes more highly expressed in long-read data (*Y* > 0) was 13 092, compared to 5671 in short-read data (*Y* < 0). No genes exhibited identical expression levels (*Y* = 0). Using a significance threshold of *P* < 0.01 and |log_10_ fold change| ≥ 2, we identified 1838 long-read-specific genes, 821 short-read-specific genes, and 2018 commonly detected genes (significant but with low fold change). Despite ~72-fold higher total read count in short-read sequencing, only 821 genes were uniquely detected by that method, whereas 1838 were uniquely identified by long-read sequencing. Notably, as shown in Supplementary Table S1, the percentage of exon–exon junction reads, which was a key indicator of transcript coverage, was substantially higher in long-read data, even though total mapped and intergenic read counts were comparable across platforms. This suggests that long-read sequencing yields more informative reads per molecule, enabling improved detection of structurally complex or low-abundance transcripts that are often missed by short-read protocols. Overall, long-read sequencing captured a broader and deeper transcriptomic landscape, including genes with higher expression levels, despite a lower overall read count. To assess consistency across individuals by generating a heatmap of log_10_-transformed TPM values (Fig. 1F). In short-read data, samples S#1 and S#2 clustered separately from S#3 and S#4, suggesting within-platform variability. In contrast, long-read samples (L#1–L#4) formed a tight cluster, indicating greater inter-individual consistency. Since the long- and short-read libraries were prepared from the same RNA samples at the same time, the observed differences are unlikely to be solely due to sequencing depth. Short-read measurements may be more susceptible to variability introduced during library preparation, whereas long-read sequencing showed more consistent transcript detection across individuals.

To evaluate the biological relevance of these platform-specific gene sets, we performed GO enrichment analysis using the differentially detected genes (Fig. 1G and Supplementary Fig. S1B). Long-read-specific genes were enriched for pathways related to immune and hematopoietic differentiation, macroautophagy and proteolysis, endoplasmic reticulum stress responses, and other nutrient- or stress-responsive processes. In contrast, short-read-specific genes showed strong enrichment for mitochondrial organization, oxidative phosphorylation, electron transport chain activity, and ATP synthesis, reflecting core metabolic and respiratory functions. Genes that were not differentially expressed between platforms were enriched for broader physiological pathways, including ion transport and membrane potential regulation (Supplementary Fig. S1B). These results highlight complementary strengths of the two sequencing platforms in capturing distinct functional layers of the blood transcriptome.

### Benchmarking long- and short-read RNA-seq against microarray reference

**Figure 2.**
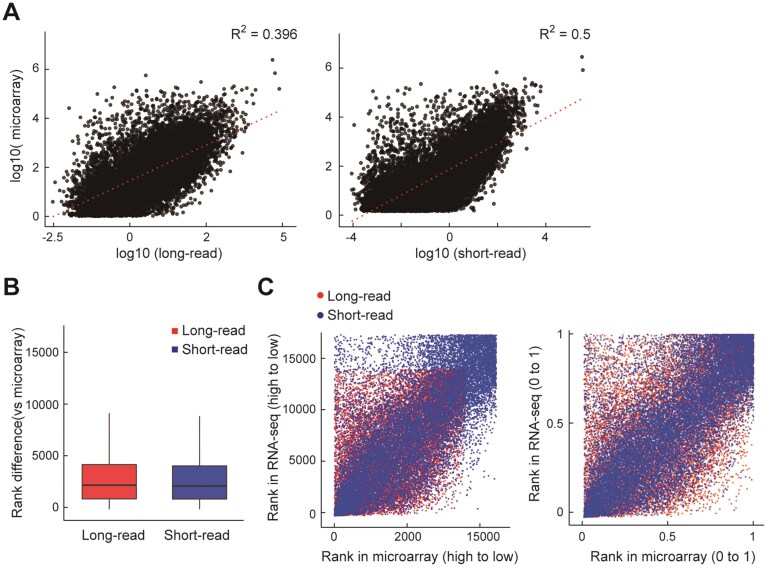
Comparison of long-read, short-read, and microarray expression data. Expression data of genes commonly detected by all three platforms (long-read RNA-seq, short-read RNA-seq, and microarray) were used for comprehensive comparisons including scatter plots, rank correlation plots, scaled rank correlation plots, and boxplots. (**A**) Scatter plots comparing gene expression levels between RNA-seq and microarray platforms. The left panel shows log_10_-transformed expression values of long-read RNA-seq (*x*-axis) versus microarray (*y*-axis), and the right panel shows log_10_-transformed short-read RNA-seq (*x*-axis) versus microarray (*y*-axis). Each dot represents a gene detected in both datasets. Red dotted lines indicate the regression line, and the coefficient of determination (*R*²) is annotated on each panel. (**B**) Rank correlation plots comparing the gene ranking (based on expression) between RNA-seq and microarray datasets. The X-axis represents gene rank in the microarray data (from highest to lowest), and the *y*-axis shows the corresponding rank in long-read (red) or short-read (blue) RNA-seq. Each dot represents a gene. Genes from short-read RNA-seq are more concentrated in the lower rank region, while those from long-read RNA-seq are more widely dispersed, reflecting differences in concordance with the microarray platform. (**C**) Scaled rank correlation plot, in which rank values for both RNA-seq and microarray were normalized between 0 and 1. This revealed comparable variability between long- and short-read RNA-seq when the number of paired genes was adjusted. (**D**) Boxplots showing the absolute rank differences between microarray and RNA-seq (long-read or short-read) for shared genes. The *y*-axis indicates the absolute difference in gene expression rank compared to microarray data. Red and blue represent long- and short-read RNA-seq, respectively. The central line within each box represents the median, while the box and whiskers indicate the interquartile range (IQR) and overall spread of the rank differences.

To evaluate the reliability of expression rankings obtained from long- and short-read RNA-seq, we compared the results with microarray analysis performed on the same RNA samples [20]. Microarrays measure gene expression using short oligonucleotide probes derived from established gene annotations, allowing sensitive detection of known transcripts. Although RNA-seq provides broader transcriptome coverage and the ability to detect novel isoforms, microarrays often show higher sensitivity for well-characterized or low-abundance genes. These methodological characteristics explain why short-read RNA-seq, which also relies on short fragments derived from poly(A)-selected transcripts, tends to show stronger concordance with microarray profiles than long-read RNA-seq. To confirm the hypothesis, a comparison of all genes commonly detected across long-read RNA-seq, short-read RNA-seq, and microarray platforms, using expression ranks (based on TPM or signal intensity) rather than absolute values as shown in Fig. 2A. Correlation analysis revealed that short-read RNA-seq exhibited a higher coefficient of determination (*R*^2^ = 0.500) against microarray data compared to long-read RNA-seq (*R*^2^ = 0.396), indicating that short-read RNA-seq more closely reflects microarray expression profiles. Figure 2B displays boxplots of the absolute rank differences between RNA-seq platforms and microarray data. The results indicate that short-read RNA-seq exhibits a slightly lower median absolute rank difference compared to long-read RNA-seq, suggesting marginally higher concordance in gene expression ranking. However, this observation likely reflects differences in gene coverage and detection sensitivity rather than fundamental discrepancies in measurement accuracy. To further investigate these rank-based relationships, we performed scaled rank correlation analyses (Fig. 2C), which confirmed a similar degree of concordance between the two RNA-seq platforms and microarray data when controlling for gene set size and expression variability. These results demonstrate that both RNA-seq platforms produce reproducible gene expression measurements that broadly agree with microarray data, with short-read RNA-seq showing a modest advantage in overall similarity. In contrast, Supplementary Figure S2 presents a filtered subset of genes selected based on expression thresholds (TPM > 2.0 for long-read RNA-seq, TPM > 0.2 for short-read RNA-seq) and statistical significance (FDR < 0.05). These thresholds were chosen to include only sufficiently expressed genes, minimizing the influence of low-abundance transcripts and measurement noise. The selected criteria are intended as practical guidelines rather than strict cutoffs for platform comparison, providing representative gene sets for illustrative purposes. Under these criteria, the number of genes detected by long-read RNA-seq was markedly greater (5539 genes) than that detected by short-read RNA-seq (1139 genes). This result may be interpreted as an advantage of long-read RNA-seq in capturing a broader range of highly expressed genes. However, this apparent superiority is primarily due to differences in coverage depth and detection sensitivity between platforms rather than a true biological difference. Therefore, such filtered gene set comparisons carry inherent bias, and the more comprehensive, unfiltered analysis shown in Fig. 2 provides a less biased and more appropriate assessment of transcriptome-wide concordance. For unbiased platform-to-platform comparison (Fig. 2), only genes detected in all three datasets (long-read, short-read, and microarray) were used. In contrast, Supplementary Figure S2 examines only the DEGs identified between long- and short-read sequencing, and evaluates how these platform-specific gene sets relate to microarray measurements. Taken together, these findings underscore the complementary nature of long- and short-read RNA-seq technologies. While short-read RNA-seq demonstrates closer agreement with microarray data, likely due to similarities in measurement principles such as probe-based detection and shorter reads, long-read RNA-seq excels in capturing a wider and more diverse set of transcripts, including full-length isoforms. Recognizing these technical differences is crucial for accurate interpretation and integration of transcriptomic data across platforms.

### Comparative transcript discovery between long- and short-read RNA-seq

**Figure 3.**
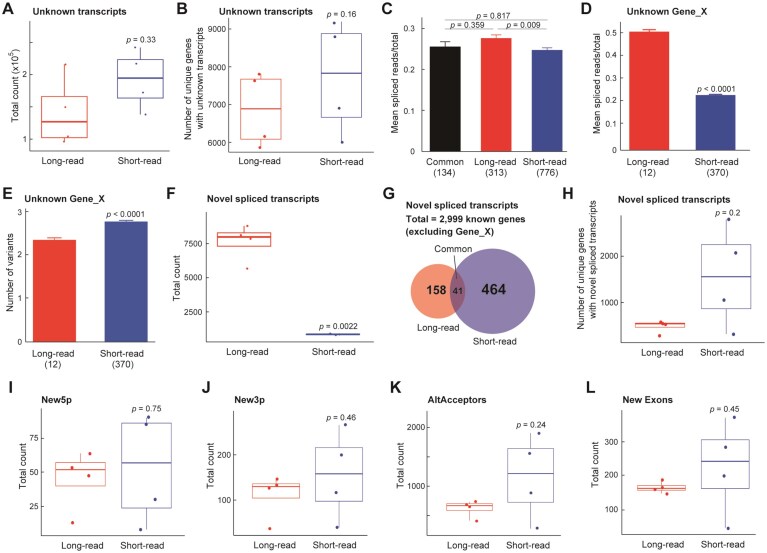
Transcript discovery analysis using long- and short-read RNA-seq. (**A**) Comparison of the number of newly identified transcripts between long-read (red) and short-read (blue) RNA-seq data (*n* = 4 per group). Box-and-whisker plots show the total counts of Unknown transcripts as defined by the CLC Genomics Workbench. Each box represents the IQR, the center line indicates the median, and whiskers extend to 1.5 × IQR. Statistical significance was assessed using a two-sided Student’s *t*-test. *P*-values are indicated on the plot. The definition of “Unknown transcripts” is referred to sequences with no annotation match in the reference database. (**B**) Box plots show the number of genes (excluding completely unannotated ones) for which at least one unknown transcript was detected. Red and blue indicate long- and short-read data, respectively. *P*-values are indicated on the plot. (**C**) Mean ratio of spliced reads to total reads for known genes that are commonly detected by both platforms (“Common”, *n* = 134), uniquely by long-read (*n* = 313) or short-read RNA-seq (*n* = 756). Bars represent the mean ± standard error (SE) of the spliced read ratio in each group. Statistical significance was assessed using one-way ANOVA followed by Tukey’s post-hoc test. A significant difference was observed between long- and short-read RNA-seq groups (*P* = 0.009), suggesting that long-read RNA-seq preferentially detects transcripts with higher splicing activity. (**D**) Comparison of the proportion of spliced reads among total reads in transcript regions uniquely detected by long-read (red) and short-read (blue) sequencing. Only non-overlapping transcript clusters (Gene_X in CLC genomics) defined by identical start and end genomic positions were analyzed. Long-read data showed a significantly higher spliced read ratio (mean = 0.50 versus 0.23, *P* < 2.2 × 10⁻^16^, Welch’s *t*-test), indicating enhanced detection of spliced transcripts. (**E**) Average number of transcript variants per Gene_X identified uniquely by long-read (red) and short-read (blue) sequencing. Each Gene_X represents a transcript cluster defined by shared start and end positions. The short-read RNA-seq could exhibit a slightly higher number of variants (mean = 2.76, SE = 0.028) than long-read (mean = 2.34, SE = 0.045), possibly due to fragmentation or over-splitting in short-read assembly. Error bars indicate standard errors of the means. (**F**) Box-and-whisker plots show the total counts of Novel spliced transcripts as defined by the CLC Genomics Workbench. Each box represents the IQR, the center line indicates the median, and whiskers extend to 1.5 × IQR. Statistical significance was assessed using a two-sided Student’s *t*-test. *P*-values are indicated on the plot. The definition of “Novel spliced transcripts” indicates putative novel isoforms of known genes. (**G**) Venn diagram showing the overlap of novel spliced transcripts detected by short-read and long-read RNA-seq. A total of 2999 novel spliced transcripts were identified from known gene loci (excluding Gene_X). Among them, 464 transcripts were detected only in short-read datasets, 158 only in long-read datasets, and 2377 were detected in at least one sample from both platforms (non-specific). Notably, 41 transcripts were consistently detected across all eight datasets (i.e. in all four individuals by both platforms) and were classified as “common.” (**H**) Comparison of novel spliced transcripts in platform specific genes. Box plots represent the number of novel 5′ ends detected per sample in genes uniquely identified by either short-read or long-read RNA-seq. No significant difference was observed between platforms (*P* = 0.753, Wilcoxon rank-sum test). (**I**) Comparison of newly identified 5′ transcript ends (New5p) in platform-specific genes. (**J**) Comparison of newly identified 3′ transcript ends (New3p) in platform-specific genes. (**K**) Comparison of alternative acceptor sites (AltAcceptors) in platform-specific genes. (**L**) Comparison of newly identified internal exons (NewExons) in platform-specific genes. Long- and short-read RNA-seq showed similar capacity in detecting novel internal exons in platform-specific genes (*P* = 0.448).

To investigate differences in transcript discovery between sequencing platforms, we first examined the number of unknown transcripts detected across four samples (Fig. 3A). For this purpose, we employed the widely used CLC Genomics transcript discovery tool to directly compare short- and long-read datasets. By using a software accessible to all researchers, we aimed to obtain objective and reproducible results. To avoid potential confusion, we first define the key transcript categories used in this chapter. “Unknown transcripts” refer to transcript clusters not annotated in current gene models (based on the T2T-CHM13 reference), “Gene_X” denotes a subset of unknown transcripts with identical start and end coordinates, and “novel spliced transcripts” indicate newly identified splicing events within known genes, where the combination of existing exons or splice sites differs from the annotated models, resulting in new RNA variants. While short-read RNA-seq identified a greater number of unknown transcripts than long-read RNA-seq, this difference was not statistically significant (*P* = 0.33, Wilcoxon test). We then quantified the number of unique genes associated with these unknown transcripts in each sample, excluding platform-specific gene clusters such as Gene_X (Fig. 3B). On average, short-read RNA-seq detected a higher number of unique genes with unknown transcripts (mean = 7764) compared to long-read RNA-seq (mean = 6634), although the difference did not reach statistical significance (*P* = 0.16, Wilcoxon rank-sum test). To further explore potential differences in splicing patterns, we calculated the mean ratio of spliced reads to total reads (spliced/total) for known genes associated with unknown transcripts (Fig. 3C). The spliced read ratio was significantly higher in the long-read-specific group than in the short-read-specific group (mean ± SEM: 0.67 ± 0.01 versus 0.64 ± 0.01, *P* = 0.009, Tukey’s HSD test), supporting the idea that long-read sequencing more effectively captures novel splicing events. The shared group showed an intermediate ratio (0.65 ± 0.01), with no statistically significant differences compared to either platform-specific group. We next focused on transcript clusters (termed Gene_X) that were uniquely identified by either long-read or short-read sequencing, defined by identical start and end coordinates. No overlapping genes were found between the two groups. The proportion of spliced reads among total mapped reads was significantly higher in the long-read group (Fig. 3D), suggesting more efficient capture of full-length spliced transcripts. To evaluate the structural complexity of transcripts within uniquely detected Gene_X regions, we quantified the number of transcript variants per Gene_X. Short-read-specific Gene_X regions exhibited a slightly higher average number of variants (mean = 2.76 ± 0.028) compared to long-read-specific regions (mean = 2.34 ± 0.045), a statistically significant difference (*P* = 5.29 × 10⁻^10^, Welch’s *t*-test) (Fig. 3E).

Next, we analyzed *novel spliced transcripts*, which represent newly identified splicing events within known gene loci (excluding Gene_X), in contrast to *unknown transcripts*, which correspond to entirely novel transcript clusters not annotated in current gene models. The total number of novel spliced transcripts was significantly higher in long-read RNA-seq compared to short-read RNA-seq (Fig. 3F, *P* = 0.002), indicating that long-read sequencing has a superior capacity to detect such novel splicing events. We than examined the overlap of novel spliced transcripts between short-read and long-read datasets. A total of 2999 transcripts were identified from known genes, of which 2377 (79.2%) were detected at least one sample in both platforms. In contrast, 464 transcripts were specific to short-read data, possibly reflecting false positives due to inference-based splicing prediction or lower coverage in long-read. Conversely, 158 transcripts were unique to long-read, likely representing complex splicing events (e.g. intron retention or multiple splice junctions) that are inherently undetectable by short-read sequencing (Fig. 3G). To assess the reproducibility and distribution of these novel splicing events across samples, we compared the number of genes containing novel spliced transcripts in each platform. Consistent with transcript-level comparisons, long-read RNA-seq tended to identify a smaller but more stable set of genes harboring novel splicing events, whereas short-read sequencing detected a larger and more variable gene set; however, the difference was not statistically significant (Fig. 3H, *P* = 0.2). We further compare the characteristics of novel splicing events detected by each sequencing platform, we categorized genes associated with these transcripts into three groups based on their detection patterns. Genes that were identified in at least one sample from both the long- and short-read datasets were defined as “non-specific,” whereas those consistently detected across all eight datasets (i.e. in all four individuals by both platforms) were defined as “common.” This classification enabled us to assess not only platform-specific transcript discovery but also the reproducibility and reliability of detection across sequencing technologies. To quantitatively assess differences in transcript structure features between sequencing platforms, we compared the number of newly identified transcript elements, specifically 5′ ends (New5p), 3′ ends (New3p), alternative acceptor sites (AltAcceptors), and novel internal exons (NewExons) between long- and short-read datasets. For transcripts detected exclusively in either long-read or short-read RNA-seq (i.e. platform-specific genes), there were no statistically significant differences between platforms in the number of newly identified 5′ ends (*P* = 0.753), 3′ ends (*P* = 0.458), alternative acceptors (*P* = 0.238), novel internal exons (*P* = 0.448), or detected gene count (*P* = 0.2) (Fig. 3I–L). These results indicate that both platforms detected a comparable number of novel transcript structures within platform-specific gene sets. Next, we focused on genes for which transcripts were detected by non-specific platforms. Again, no significant differences were observed in the number of newly identified transcript elements: New5p (*P* = 0.268), New3p (*P* = 0.464), AltAcceptors (*P* = 0.305), and NewExons (*P* = 0.716). The overall gene count was also not significantly different (*P* = 0.2) (Supplementary Fig. S3A–D). Finally, we examined transcript elements within common genes that were consistently detected across both platforms (“common” gene set). The number of newly identified 3′ ends (*P* = 0.128), alternative acceptors (*P* = 0.456), and internal exons (*P* = 0.426) did not differ significantly between platforms. However, the number of novel 5′ ends showed a wide distribution with no statistically significant difference (*P* = 1.0) (Supplementary Fig. S3E–H). The gene count was not evaluated in this group due to lack of inter-platform variability. In contrast, Gene_X transcripts, defined as novel transcript clusters not overlapping with any known genes, showed platform-dependent detection with 416 long-read-specific, 1416 short-read-specific, and only 9 commonly identified regions. Due to the inherent differences in transcript assembly and annotation, overlap of Gene_X transcripts was evaluated separately.

In summary, long-read RNA-seq demonstrated a superior ability to detect novel splicing events and capture complete spliced transcript structures, particularly within known gene loci. While both platforms showed comparable performance in identifying structural transcript features within platform-specific genes, overlap between platforms was limited, especially for entirely novel transcript clusters (Gene_X). Importantly, this comprehensive transcriptome analysis revealed a subset of consistently detected novel transcripts across all samples and platforms—41 novel spliced transcripts within known genes and 9 entirely new transcript clusters (Gene_X)—highlighting the potential biological relevance and reproducibility of these discoveries. These findings underscore not only the technical complementarity of long- and short-read platforms, but also their combined utility in uncovering previously unannotated yet consistently expressed transcript variants.

### Comparative analysis of fusion transcript detection by long- and short-read RNA-seq using FusionGDB2

**Figure 4.**
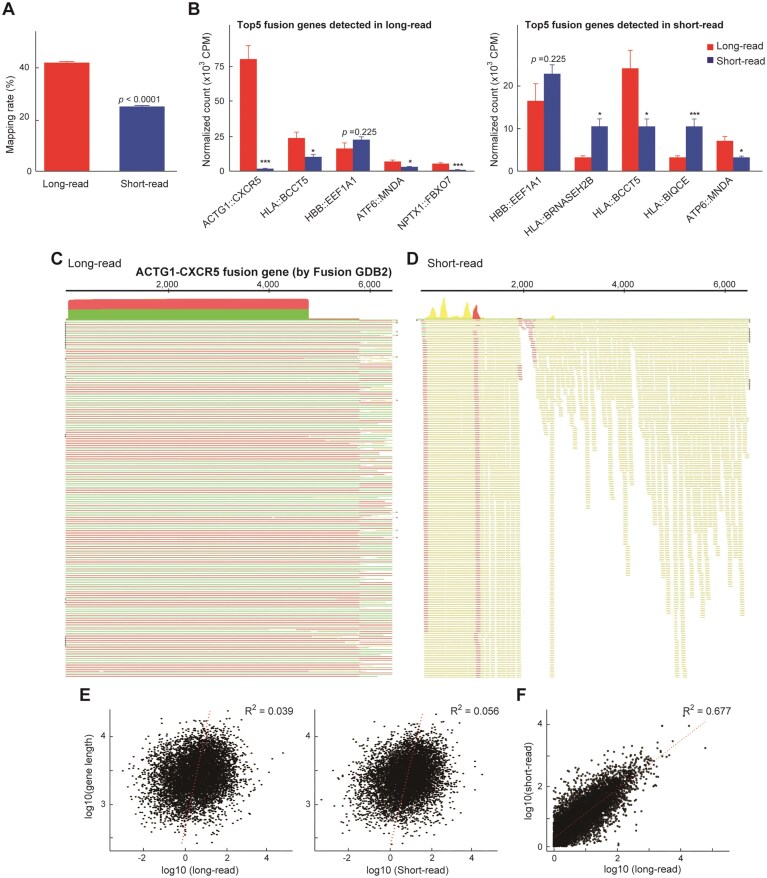
Fusion gene analysis using long- and short-read RNA-seq. (**A**) Mapping rate (%) of fusion transcripts based on FusionGDB2 reference (*n* = 65 536). Long-read data showed a significantly higher mapping rate than short-read data (Welch’s *t*-test, *P* = 6.56 × 10⁻^8^). Error bars indicate standard error (SE). (**B**) Top 5 fusion transcripts with the highest in long- and short-read datasets. Read counts were normalized to the number of mapped reads per sample, then averaged across four individuals for each platform. Long- and short-read values for each fusion gene are shown side-by-side. Top 5 fusion genes in long-read (left) and short-read (right). (**C**) Representative long-read RNA-seq alignments at the ACTG1::CXCR5 fusion junction. Reads show continuous, high-quality mapping, with red indicating forward strand and green indicating reverse strand sequencing, spanning the fusion site. This demonstrates full-length coverage and high-confidence detection of the fusion transcript. (**D**) Representative short-read RNA-seq alignments at the ACTG1::CXCR5 fusion junction. The mapping is fragmented with numerous mismatches highlighted in yellow, and reads fail to fully span the fusion breakpoint, indicating limited detection capability for this complex fusion transcript by short-read sequencing. (**E**) Scatter plots showing the correlation between fusion transcript length and normalized read count in long- and short-read data (log_10_ scale). While the correlation was statistically significant (*P* < 0.001), the explanatory power was limited (*R*^2^ = 0.056 for short-read and 0.039 for long-read). Regression lines are shown in red. (**F**) Correlation plot comparing normalized expression levels of fusion genes detected by short-read and long-read RNA-seq platforms. The strong positive correlation (*R*^2^ = 0.67) indicates a high degree of concordance between the two sequencing methods in detecting fusion transcripts across the dataset.

Gene fusions represent critical oncogenic drivers and biomarkers in various malignancies, underscoring their clinical and biological significance. The advent of RNA-seq technologies has facilitated their detection; however, disparities in sequencing read length and platform-specific biases influence the sensitivity and specificity of fusion transcript identification. In recent years, the detection of gene fusions has increasingly shifted from short-read to long-read RNA-seq [18, 27], which provide full-length transcript structures, thereby improving the accuracy of fusion event detection. However, to our knowledge, there are no studies directly comparing fusion gene detection between short-read and long-read sequencing using the same RNA samples. This study systematically evaluates the differential detection capacity of fusion transcripts between long- and short-read RNA-seq approaches, leveraging the extensive FusionGDB2 fusion gene repository. We compared the fusion transcript mapping rates between long- and short-read RNA-seq data using FusionGDB2 (ver. 2024-12, 65 536 reference fusions) [24]. The mapping rate was significantly higher in long-read samples compared to short-read samples (Welch’s *t*-test, *P* = 6.56 × 10^–8^), indicating that long-read RNA-seq captures more fusion transcript signals (Fig. 4A). Among the 7946 fusion genes detected using the FusionGDB2 reference, two were uniquely identified by the long-read platform, 211 were uniquely identified by the short-read platform and 7733 fusion genes were detected by both. This indicates a high degree of concordance between long- and short-read platforms in terms of fusion gene detection, with minimal platform-specific bias. To further assess platform-specific detection patterns, we extracted the top five fusion transcripts with the highest normalized expression levels from each dataset (Fig. 4B). Although some fusion transcripts overlapped between the two lists, their relative abundance profiles were generally consistent across platforms. To visualize this trend, we presented all selected transcripts using the same scale based on the long-read dataset, which revealed broadly similar detection patterns regardless of the sequencing method. However, the fusion transcript *ACTG1–CXCR5* was detected at markedly higher levels in long-read RNA-seq compared to short-read data, with CPM values exceeding 75 000. Visual inspection of the mapping results revealed full-length and high-confidence alignment across the fusion junction in long-read reads (Fig. 4C), while short-read mapping was fragmented and failed to span the fusion site (Fig. 4D). These results highlight the potential underestimation of fusion transcripts using short-read sequencing alone, particularly for complex or long fusion events. In addition, we investigated whether the length of fusion transcripts affected detection efficiency across platforms. Correlation analysis between transcript length and normalized read counts demonstrated statistically significant but weak associations (*R*^2^ = 0.056 for short-read, *R*^2^ = 0.039 for long-read; *P* < 0.001 for both), suggesting that transcript length had minimal impact on fusion detection by either sequencing method (Fig. 4E). Figure 4F shows the correlation plot of detected fusion genes between short-read and long-read sequencing methods, revealing a strong positive correlation with an *R*^2^ value of 0.67, indicating high concordance between the two platforms. Thus, our results highlight the critical need to consider sequencing platform biases in fusion gene studies and support the integration of long-read RNA-seq for improved detection fidelity.

### Platform-specific miRNA-target gene networks and associated protein interaction structures

**Figure 5.**
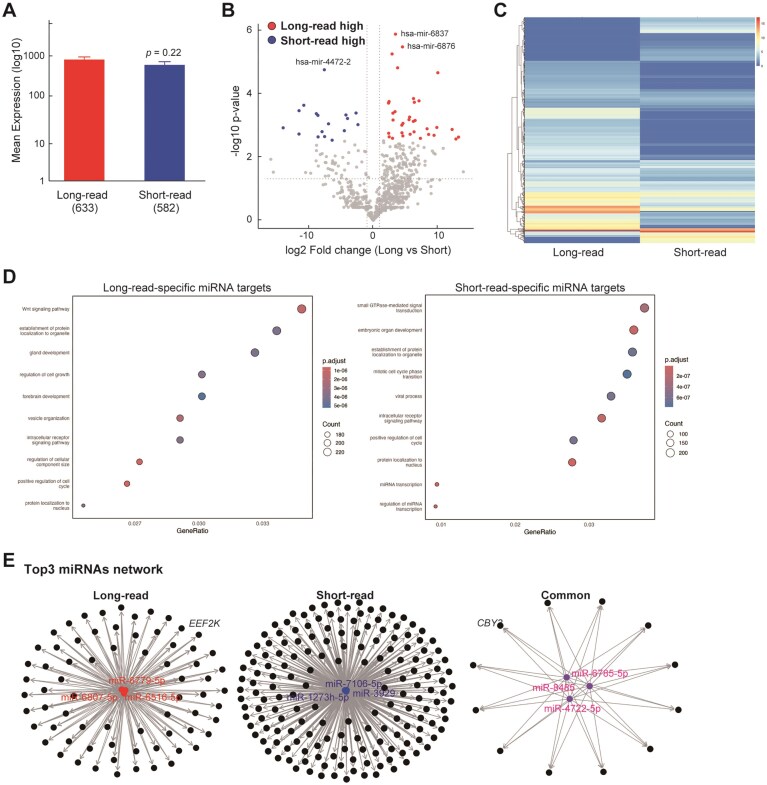
pri-miRNA analysis using long- and short-read RNA-seq. (**A**) Comparison of average pri-miRNAs expression levels detected by long- and short-read RNA-seq platforms. The bar graph shows the mean expression (TPM) ± standard error (SE) on a log_10_ scale for each platform. Although the mean expression was higher in the long-read dataset compared to the short-read dataset, Welch’s *t*-test indicated no significant difference (*P* = 0.215). (**B**) Volcano plot showing differential expression of pri-miRNAs between long- and short-read platforms. Red dots indicate pri-miRNAs significantly upregulated in the long-read dataset (*n* = 34), while blue dots indicate pri-miRNAs significantly upregulated in the short-read dataset (*n* = 17). Gray dots represent pri-miRNAs without significant differential expression. The plot highlights distinct subsets of pri-miRNAs preferentially detected by each platform. (**C**) Heatmap of significantly differentially expressed pri-miRNAs (adjusted *P*-value < 0.05 and average TPM > 10) between long- and short-read platforms. Expression levels are shown in log_2_-transformed TPM values across samples, illustrating distinct expression patterns for pri-miRNAs enriched in either platform. (**D**) GO enrichment (Biological Process) results based on target genes of mature miRNAs corresponding to the differentially expressed pri-miRNAs (long versus short). (**E**) Mature miRNA-target gene network analysis showing shared target genes regulated by the top 3 mature miRNAs predicted from differentially expressed pri-miRNAs detected in each platform (long-read, short-read) and common pri-miRNAs. Each black node represents a unique target gene; miRNA nodes are colored (red for long-read, blue for short-read, purple for common). Node labels are omitted for clarity due to network complexity.

miRNAs play pivotal regulatory roles in cancer and other diseases. During our transcriptome annotation, we observed that several unannotated or extended regions corresponded to known miRNA loci, prompting us to investigate the detectability and expression of their primary transcripts (pri-miRNAs) across sequencing platforms. For clarity, we use the term “pri-miRNA” when referring to the primary transcript detected in RNA-seq data, and “miRNA” when discussing the mature functional form registered in miRBase v2.0 and used for target prediction and network analysis. Although multiple mature miRNAs can arise from a single pri-miRNA, this analysis treats each pri-miRNA as a single unit for downstream inference. It should be noted that rigorous quantification of mature miRNAs typically requires small RNA-specific library preparation, and the current study’s comparison between long- and short-read RNA-seq is therefore limited in this regard. However, the fragmentation-free nature of long-read sequencing might enhance detection of full-length pri-miRNAs, providing more faithful reconstruction of transcript structures. In total of 816 pri-miRNAs were detected across all samples, where detection was defined as presence in at least one of the four replicates per platform. Among these, 234 pri-miRNAs (28.7%) were uniquely detected by the long-read platform, 183 pri-miRNAs (22.4%) uniquely detected by the short-read platform, and 399 pri-miRNAs (48.9%) were commonly detected by both platforms under this permissive criterion. For these 399 commonly detected pri-miRNAs, expression levels showed a strong positive correlation between platforms (Pearson *r* = 0.85, *P* < 0.001), indicating high quantification consistency (Supplementary Fig. S4A). Applying a stricter criterion requiring detection across all four replicates per platform reduced the overlap: 158 pri-miRNAs were uniquely detected by the long-read platform, 94 by the short-read platform, and 102 were shared (Supplementary Data S1). These results suggest that while overall detection is largely overlapping, reproducibility across replicates differs between platforms. Figure 5A compares average pri-miRNA expression levels between the platforms, showing no statistically significant difference by Welch’s *t*-test (*P* = 0.215). Differential expression analysis identified 34 pri-miRNAs significantly upregulated in the long-read dataset and 17 in the short-read dataset (adjusted *P* < 0.05, average TPM > 10; Fig. 5B). These differences are further illustrated in the heatmap (Fig. 5C), highlighting platform-specific expression profiles and reinforcing the complementary nature of both sequencing technologies.

To explore potential functional consequences of these expression differences, we inferred mature miRNAs from the differentially expressed pri-miRNAs (as annotated in miRBase) and predicted their target genes. GO enrichment analysis revealed that targets of long-read-specific miRNAs were enriched in terms such as Wnt signaling, protein localization to organelle, gland development, and cell growth (Fig. 5D). In contrast, short-read-specific miRNAs were associated with small GTPase-mediated signal transduction, embryonic organ development, and protein localization, suggesting some functional divergence. Targets of miRNAs derived from commonly detected pri-miRNAs (Supplementary Fig. S4B) were enriched for overlapping categories such as cell adhesion and Wnt signaling, reflecting shared regulatory themes despite platform-specific biases. We further constructed miRNA-target gene networks using mature miRNAs derived from the top-expressed pri-miRNAs in each platform, incorporating validated and predicted interactions from miRNet [28]. Networks were generated for the top 1, 2, and 3 mature miRNAs per group (long-read-specific, short-read-specific, and common). Beyond the top three, no shared target genes were observed, suggesting increased functional heterogeneity among lower-ranked miRNAs. Figure 5E displays the network architectures of the top 3 miRNAs for each platform, and Supplementary Fig. S4C shows node-labeled versions for detailed inspection. Quantitatively, the top three long-read miRNAs (miR-6779-5p, miR-6516-5p, and miR-6807-5p) collectively targeted 679, 658, and 605 genes, respectively, with 70 shared targets. The top short-read miRNAs (miR-7106-5p, miR-1273h-5p, and miR-3929) targeted 801, 671, and 513 genes, sharing 187. Common-detected miRNAs (miR-8485, miR-6785-5p, and miR-4722-5p) showed broader target profiles (906, 842, and 612), but only 12 genes were shared among them. These patterns suggest that long-read-specific miRNAs may regulate more coherent gene sets, whereas short-read-specific miRNAs exhibit broader but more dispersed target networks. Although our study used total RNA-seq rather than small RNA-enriched libraries, the fragmentation-free nature of long-read sequencing may enhance detection of full-length pri-miRNAs and allow more faithful reconstruction of mature miRNA profiles. Nonetheless, future validation using small RNA-specific protocols is needed. Taken together, our findings indicate that long- and short-read sequencing platforms offer complementary strengths in miRNA transcript detection and regulatory inference, with long-read approaches providing unique advantages in capturing the structural context of non-coding RNAs.

### Comprehensive comparison of immunoreceptor repertoires between long- and short-read RNA-seq

**Figure 6.**
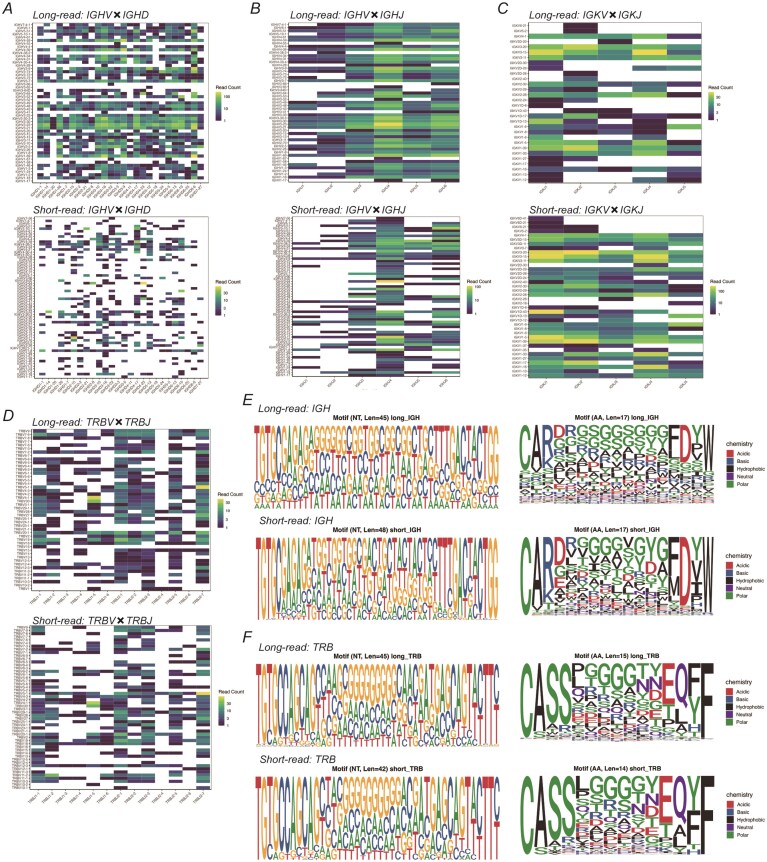
Comparison of BCR and TCR repertoires between long- and short-read sequencing using MiXCR. (**A**) Heatmap of IGHV versus IGHD gene combinations. Upper panel: long-read; Lower panel: short-read. Colors indicate read counts from yellow (high) through green to dark blue (low). (**B**) Heatmap of IGHV versus IGHJ gene combinations, with the same format as in (A). (**C**) Heatmap of IGKV versus IGKJ gene combinations. (**D**) Heatmap of TRAV versus TRAJ gene combinations. (**E**) CDR3 motif logos of IGH chains. Left: nucleotide sequences; Right: amino acid sequences. Upper panels: long-read; Lower panels: short-read. In the amino acid logos, residues are color-coded by chemical properties: acidic (red), basic (blue), hydrophobic (black), neutral, and polar (green). (**F**) CDR3 motif logos of TRB chains, shown in the same format as in (E). Repertoire diversity for each chain was evaluated using Shannon and Simpson diversity indices, as described in the main text (see Supplementary Table S2 for numerical values). Diversity indices in A,B; Long-read: Shannon = 8.592, Simpson = 0.999, Short-read: Shannon = 5.888, Simpson = 0.994. C: Long-read: Shannon = 6.125, Simpson = 0.996. Short-read: Shannon = 6.566, Simpson = 0.996. D: Long-read: Shannon = 6.640, Simpson = 0.997, Short-read: Shannon = 6.252, Simpson = 0.9973.

To clarify how long- and short-read RNA-seq differ in their ability to capture immunoreceptor repertoires, we conducted a systematic comparison of V(D)J gene usage, V-J recombination patterns, and CDR3 motif structures across both BCR and TCR chains (Fig. 6). This framework allowed us to evaluate platform-dependent differences in recombination profiles and clonal architecture, using IGH/IGK/IGL and TRA/TRB repertoires as representative examples. Despite a lower alignment rate overall, long-read sequencing exhibited a markedly higher complete CDR3 recovery rate (95.9%) compared to short-read sequencing (23.5%), as determined using MiXCR [26]. Although the overall alignment rate for long-read RNA-seq was lower than that of short-read data, the higher complete CDR3 recovery rate likely reflects the advantage of capturing full-length transcripts, which allows for more accurate reconstruction of V(D)J recombination events. The number of IGH transcripts was comparable between the two platforms (20 372 for long-read versus 17 330 for short-read). In contrast, short-read sequencing recovered more IGK/IGL and TCR (TRA and TRB) transcripts, although caution is warranted when interpreting TCR results from short-read data due to potential assembly limitations. In a quantitative comparison using CDR3 sequence-level analysis, a total of 8379 unique CDR3 sequences were identified across both platforms, with only 216 CDR3s shared between them, resulting in a low Jaccard index of 0.0258. This minimal overlap highlights the methodological differences in repertoire capture between long- and short-read sequencing. Clonal diversity was evaluated using the Shannon and Simpson diversity indices, which account for both the richness and distribution of clonotypes within a repertoire. The Shannon diversity index, which quantifies both the richness and evenness of clonotypes [29], increases with more diverse and evenly distributed repertoires. The Simpson diversity index, which measures the probability that two randomly selected reads belong to different clonotypes [29], yields values closer to one when diversity is high and dominance by particular clones is low. In our dataset, the IGH chain in the long-read data exhibited the highest clonal diversity (Shannon = 8.59; Simpson = 0.999), whereas TRD and TRG chains under short-read conditions showed substantially lower diversity (e.g. TRD: Shannon = 1.63; Simpson = 0.742). These findings suggest that long-read sequencing better captures a broader and more even clonal landscape, particularly for highly diverse or underrepresented chains such as IGH, TRD, and TRG (Supplementary Table S2). In immunoglobulin BCR repertoires, IGHV diversity was higher in long-read (Fig. 6A and B), while short-read captured a greater number of IGKV and IGLV gene combinations (Fig. 6C, and Supplementary Fig. S5A and B). But many of these corresponded to similar or overlapping CDR3 sequences, suggesting that fragmentation and assembly limitations may inflate the apparent V gene diversity without necessarily improving unique clonotype resolution. Consistent with this, amino acid motif analyses revealed that only IGHV showed substantial differences between platforms, whereas IGLV and IGKV motifs were broadly similar (Fig. 6E, and Supplementary Fig. S5D and E). This discrepancy suggests that although short-read sequencing detects more V gene types, many of these may correspond to similar or redundant CDR3 sequences, potentially reflecting annotation artifacts or lower-resolution V gene assignment. For TCR repertoires, TRBV diversity was slightly higher in the long-read data, despite shorter transcript lengths (Fig. 6D), which is consistent with higher clonotype resolution inferred from diversity indices such as Shannon entropy. Nonetheless, TRBV amino acid motifs remained largely consistent between platforms (Fig. 6F), indicating similar clonal structures. Similarly, TRAV–TRAJ combinations were more diverse in long-read data (Supplementary Fig. S5C), while motif complexity at the amino acid level was comparable (Supplementary Fig. S5F). These results indicate that long-read sequencing provides enhanced resolution at the gene recombination level, whereas clonal composition inferred from CDR3 motifs remains largely preserved between platforms.

## Discussion

In this study, we systematically compared transcriptomic profiles obtained via long- and short-read RNA-seq across multiple facets, including gene expression (Figs 1 and 2), transcript variant and novel gene detection (Fig. 3), microRNA identification (Fig. 4), fusion gene discovery (Fig. 5), and immune repertoire analysis (Fig. 6). Our findings demonstrate that long-read sequencing affords superior coverage and sensitivity in detecting a broader spectrum of transcripts, encompassing novel and functionally significant genes that are underrepresented in short-read datasets. Notably, long-read data facilitated more comprehensive and consistent identification of gene isoforms, lncRNAs, and fusion transcripts. While both platforms identified largely overlapping microRNA species, long-read sequencing captured additional microRNA variants and complex isoforms, underscoring its advantages for non-coding RNA analyses. In immune repertoire profiling, long-read sequencing enabled more precise reconstruction of full-length V(D)J recombination sequences and CDR3 motifs, particularly within immunoglobulin heavy chains, thereby enhancing clonotype resolution. Conversely, short-read sequencing yielded comparable results for select TCR chains but exhibited limitations in fully capturing repertoire diversity. Collectively, these results emphasize the complementary strengths of both sequencing technologies and advocate for their integrated application to achieve a comprehensive characterization of transcriptomic complexity, with significant implications for disease research and biomarker discovery. From a practical standpoint, long-read sequencing is not expected to replace short-read platforms in large-scale cohorts due to cost and throughput constraints. Instead, our findings support a discovery-validation division of labor, in which long-read sequencing is used for structural discovery and short-read or targeted assays are subsequently deployed for scalable screening.

Short-read RNA-seq has long been regarded as the gold standard for transcriptome profiling due to its high accuracy, cost-effectiveness, and compatibility with established expression analysis pipelines [1]. Indeed, previous studies have reported strong agreement between short-read RNA-seq and microarray results, further supporting its reliability for gene expression quantification [30–32]. Previous studies comparing short-read RNA-seq with microarray technologies have consistently reported high overall correspondence, particularly for moderately to highly expressed genes, generally in the range of 0.6–0.85 [32]. A clinical sample study further reported 0.76 concordance, with improvements via GC-content correction [31]. In our study, short-read RNA-seq similarly showed a higher correlation with microarray data (*R*^2^ = 0.500) compared to long-read RNA-seq (*R*^2^ = 0.396), which is consistent with these previously reported benchmarks. The slightly stronger alignment of short-read data may reflect methodological similarities both rely on detection of short fragments, tend to emphasize highly expressed transcripts, and underrepresent isoform complexity or low-abundance RNA species. Consistent with this interpretation, our analysis confirmed that microarray measurements rely on short oligonucleotide probes designed from established gene annotations, most of which were originally derived from short-read sequencing data. As a result, microarray signal intensities aligned more closely with short-read RNA-seq than with long-read RNA-seq, which captures full-length transcripts and reveals additional isoform diversity that microarray probes cannot detect. Therefore, the microarray comparison in Fig. 2 should be interpreted as a technical benchmark of rank concordance between platforms, rather than as a reference for biological ground truth.

Recent advances in long-read sequencing technologies, such as PacBio and Oxford Nanopore, have revealed that short-read platforms may underestimate transcript diversity and miss biologically relevant isoforms or gene variants [2, 15, 18]. These limitations are particularly evident in immune-related and non-coding transcripts that are often fragmented or misaligned in short-read data. To our knowledge, this is the first study to perform a direct, multi-platform comparison of long-read, short-read, and microarray-based transcriptome profiling using identical RNA samples from peripheral blood mononuclear cells. Despite about 72-fold difference in read depth, long-read RNA-seq demonstrated strong concordance with short-read data in both absolute expression levels (*R*^2^ = 0.771) and gene ranking (*R*^2^ = 0.834). These results confirm that long-read RNA-seq can provide reproducible expression profiles comparable to conventional methods. While short-read RNA-seq showed a higher correlation with microarray data (*R*^2^ = 0.500) than long-read RNA-seq (*R*^2^ = 0.396), this result is likely attributable to methodological similarities. Both microarray and short-read sequencing rely on probe-based or short-fragment detection, which may limit transcript resolution and underestimate full-length isoforms. In contrast, long-read sequencing captures complete transcript structures, including low-abundance or structurally complex transcripts, leading to broader transcriptome coverage. Notably, gene ontology enrichment analysis revealed that long-read-specific transcripts were associated with diverse and biologically significant processes, including innate immune responses, cell cycle regulation, and autophagy-categories that were absent from the short-read-specific gene sets. This observation suggests that long-read RNA-seq not only increases transcript detection but also enhances functional insight into complex biological systems.　

Specifically, long-read sequencing showed a significantly higher ratio of spliced reads among platform-specific unknown transcripts, supporting its improved ability to capture complete and novel splicing events (Fig. 3C and H). Although short-read RNA-seq detected more total unknown transcripts, these may partly reflect false positives from inference-based reconstruction or coverage biases. The long-read platform also identified significantly more novel spliced transcripts from known gene loci, and those transcripts exhibited higher completeness, indicating greater structural fidelity. It is important to note that the detection of transcript boundaries such as 5′ and 3′ ends in our study reflects algorithm-derived inferences generated by the CLC transcript discovery workflow, rather than experimental end-capture. Accurate mapping of native transcript termini is strongly protocol-dependent. For example, standard poly(A)-selected short-read RNA-seq does not preserve 5′ ends due to RNA fragmentation and priming strategies, whereas CAP-trapping approaches such as CAP-Trap-seq enable precise identification of 5′ cap sites [5, 33]. Likewise, methods such as FLAM-seq accurately define poly(A) tail-associated 3′ ends [34]. Because our libraries were prepared using poly(A)-selected total RNA for both long- and short-read platforms, the transcript structures reported in this study should be interpreted as computationally inferred boundaries rather than experimentally validated termini. Recognizing these protocol-dependent constraints is essential for accurate interpretation of the structural differences observed between platforms.

In the context of fusion gene detection, long-read RNA-seq achieved higher mapping rates against a comprehensive fusion reference database (FusionGDB2), with full-length, high-confidence alignment across fusion junctions; an area where short-read mapping often failed due to fragmented reads (Fig. 4C and D). While the overall concordance between platforms was high, long-read sequencing uniquely identified fusions like *ACTG1::CXCR5*, which were poorly represented or undetectable in short-read data due to read length limitations. Taken together, these findings reinforce the notion that long-read RNA-seq is particularly well-suited for the accurate detection of novel splicings, full-length transcripts, and complex gene fusions, complementing short-read data. Although long-read sequencing inherently reduces assembly-derived artifacts, false positives remain a known concern, especially for low-abundance transcripts. In this study, we required multi-evidence support (per-sample recurrence, splice-site consensus, and database alignment when available) to mitigate potential artifacts and ensure biological plausibility. Importantly, a subset of transcripts including 41 novel splice variants and 9 novel gene clusters were reproducibly detected across all individuals and platforms, underscoring the biological validity of these discoveries. As transcriptome profiling moves toward greater resolution and functional interpretation, integrating long-read RNA-seq into standard pipelines will be essential for uncovering the full complexity of gene regulation. Similar advantages of long-read sequencing in isoform resolution, fusion transcript detection, and immune-repertoire reconstruction have also been reported in studies of solid tumors, supporting the generalizability of our platform-dependent observations [18, 35].

While our study used total RNA-seq libraries for both long- and short-read platforms, it is important to note that specialized library preparation kits are commercially available for both miRNA and CDR3 region profiling. For miRNA analysis, protocols incorporating gel-based size selection or capture-based enrichment have demonstrated greater detection accuracy and reduced bias, as highlighted by recent benchmarking studies [36]. Similarly, CDR3-targeted methods, often involving multiplex PCR panels or hybrid capture, focus sequencing throughput precisely on rearranged immunoreceptor regions, providing higher clonotype resolution than bulk methodologies. In contrast, our study applied untargeted, full-length long-read RNA-seq to probe both miRNA and immune repertoires from total RNA without size selection or amplicon enrichment. While this enabled a holistic profiling of transcriptome-wide features, its performance relative to dedicated small RNA or CDR3-specific kits remains uncertain. Full-length approaches theoretically improve V(D)J reconstruction and isoform coverage, as supported by comparative studies demonstrating the structural completeness afforded by long-read sequencing [37] and comparative frameworks distinguishing CDR3-focused versus full-length analyses [38] . Notably, despite the absence of targeted enrichment, long-read sequencing enabled the reconstruction of full-length immunoglobulin and TCR transcripts, with accurate identification of CDR3 motifs and V(D)J recombination sequences, particularly within IGH chains (Fig. 6). This suggests that long-read RNA-seq holds promise for capturing immune repertoire diversity even from total RNA of peripheral blood samples. Such ability is particularly relevant for immunological studies, as rare but functionally critical clones including memory T and B cells can have profound biological significance despite their low abundance. The capacity to detect these transcripts without amplification-based bias represents an asset for understanding adaptive immune dynamics and may inform future diagnostic or prognostic applications.

Future validation studies should involve direct benchmarking of long-read data against specialized miRNA-seq and CDR3 profiling kits to assess sensitivity, clonotype resolution, and biological fidelity in detecting small and rearranged RNA species. Recent advances in transcriptome analysis have led to the widespread application of RNA-seq in forensic and clinical pathology settings, particularly using formalin-fixed, paraffin-embedded (FFPE) tissues [39–41]. These samples are inherently degraded and fragmented, rendering short-read sequencing the only feasible approach due to its compatibility with fragmented RNA molecules. In such contexts, the use of full-length long-read sequencing offers limited technical advantage, as the input RNA is no longer intact and full-length transcript reconstruction becomes unattainable. Conversely, the unique strength of long-read RNA-seq lies in its ability to capture full-length transcripts, novel isoforms, and fusion events from high-quality or fresh-frozen samples [13, 18]. These capabilities make it particularly valuable in exploratory studies where previously uncharacterized transcript structures are being discovered. Importantly, once such transcript variants or fusion genes are identified in a small cohort using long-read sequencing, targeted primers can be designed for downstream validation via RT-qPCR or digital PCR across large clinical cohorts including FFPE-derived samples. This pipeline circumvents the need for large-scale short-read RNA-seq once specific targets have been defined, streamlining validation in retrospective bio-banked samples. Therefore, while long-read RNA-seq is not always applicable in degraded clinical specimens, it plays a foundational role in transcript discovery, after which focused, cost-effective validation strategies can be deployed in large FFPE-based cohorts.

## Supplementary Material

ugag006_Supplemental_Files

## Data Availability

The raw sequencing data (FASTQ files) generated from both long- and short-read RNA-seq are not publicly available due to ethical and privacy considerations. Given the small sample size (*n* = 4) and the possibility of identifying individuals through additional analyses such as variant calling, we are unable to share the primary sequencing data in compliance with patient confidentiality policies. However, processed data, including transcript-level TPM values and differential expression results, are provided as Supplementary Data to support reproducibility and further analysis.

## References

[1] Stark R, Grzelak M, Hadfield J. RNA-seq: the teenage years. Nat Rev Genet. 2019;20:631–56. 10.1038/s41576-019-0150-2. 31341269

[2] Chen Y, Davidson NM, Wan YK et al. A systematic benchmark of Nanopore long-read RNA sequencing for transcript-level analysis in human cell lines. Nat Methods. 2025;22:801–12. 10.1038/s41592-025-02623-4. 40082608 PMC11978509

[3] Inamo J, Suzuki A, Ueda MT et al. Long-read sequencing for 29 immune cell subsets reveals disease-linked isoforms. Nat Commun. 2024;15:4285. 10.1038/s41467-024-48615-4. 38806455 PMC11133395

[4] Reese F, Williams B, Balderrama-Gutierrez G et al. The ENCODE4 long-read RNA-seq collection reveals distinct classes of transcript structure diversity. bioRxiv. 2023; 16 May 2023, preprint: not peer reviewed.

[5] Pardo-Palacios FJ, Wang D, Reese F et al. Systematic assessment of long-read RNA-seq methods for transcript identification and quantification. Nat Methods. 2024;21:1349–63. 10.1038/s41592-024-02298-3. 38849569 PMC11543605

[6] Jain M, Koren S, Miga KH et al. Nanopore sequencing and assembly of a human genome with ultra-long reads. Nat Biotechnol. 2018;36:338–45. 10.1038/nbt.4060. 29431738 PMC5889714

[7] Wick RR, Judd LM, Holt KE. Performance of neural network basecalling tools for Oxford Nanopore sequencing. Genome Biol. 2019;20:129. 10.1186/s13059-019-1727-y. 31234903 PMC6591954

[8] Wenger AM, Peluso P, Rowell WJ et al. Accurate circular consensus long-read sequencing improves variant detection and assembly of a human genome. Nat Biotechnol. 2019;37:1155–62. 10.1038/s41587-019-0217-9. 31406327 PMC6776680

[9] Tardaguila M, de la Fuente L, Marti C et al. SQANTI: extensive characterization of long-read transcript sequences for quality control in full-length transcriptome identification and quantification. Genome Res. 2018;28:396–411. 10.1101/gr.222976.117. 29440222 PMC5848618

[10] Wyman D, Mortazavi A. TranscriptClean: variant-aware correction of indels, mismatches and splice junctions in long-read transcripts. Bioinformatics. 2019;35:340–2. 10.1093/bioinformatics/bty483. 29912287 PMC6329999

[11] Mestre-Tomas J, Liu T, Pardo-Palacios F et al. SQANTI-SIM: a simulator of controlled transcript novelty for lrRNA-seq benchmark. Genome Biol. 2023;24:286. 10.1186/s13059-023-03127-0. 38082294 PMC10712166

[12] Budnick A, Franklin MJ, Utley D et al. Long- and short-read sequencing methods discover distinct circular RNA pools in Lotus japonicus. Plant Genome. 2024;17:e20429. 10.1002/tpg2.20429. 38243772 PMC12806940

[13] Begum G, Albanna A, Bankapur A et al. Long-read sequencing improves the detection of structural variations impacting complex non-coding elements of the genome. Int J Mol Sci. 2021;22:2060. 10.3390/ijms22042060. 33669700 PMC7923155

[14] Yang Y, Yang R, Kang B et al. Single-cell long-read sequencing in human cerebral organoids uncovers cell-type-specific and autism-associated exons. Cell Rep. 2023;42:113335. 10.1016/j.celrep.2023.113335. 37889749 PMC10842930

[15] Wang M, Li Y, Wang J et al. Integrating short-read and long-read single-cell RNA sequencing for comprehensive transcriptome profiling in mouse retina. Genome Res. 2025;35:740–54. 10.1101/gr.279167.124. 40050124 PMC12047235

[16] Vellas C, Doudou A, Mohamed S et al. Comparison of short-read and long-read next-generation sequencing technologies for determining HIV-1 drug resistance. J Med Virol. 2024;96:e29951. 10.1002/jmv.29951. 39387352

[17] Zajac N, Zhang Q, Bratus-Neuenschwander A et al. Comparison of single-cell long-read and short-read transcriptome sequencing via cDNA molecule matching: quality evaluation of the MAS-ISO-seq approach. NAR Genom Bioinform. 2025;7:lqaf089. 10.1093/nargab/lqaf089. 40630932 PMC12231600

[18] Ament IH, DeBruyne N, Wang F et al. Long-read RNA sequencing: a transformative technology for exploring transcriptome complexity in human diseases. Mol Ther. 2025;33:883–94. 10.1016/j.ymthe.2024.11.025. 39563027 PMC11897757

[19] Okada H, Nasti A, Sakai Y et al. Evaluation of long-read RNA sequencing procedures for novel isoform identification and quantification in Human whole blood. Genes. 2025;16:1075. 10.3390/genes16091075. 41010020 PMC12469794

[20] Sakai Y, Nasti A, Takeshita Y et al. Eight-year longitudinal study of whole blood gene expression profiles in individuals undergoing long-term medical follow-up. Sci Rep. 2021;11:16564. 10.1038/s41598-021-96078-0. 34400700 PMC8368195

[21] Liu CH, Di YP. Analysis of RNA sequencing data using CLC Genomics Workbench. Methods Mol Biol. 2020;2102:61–113. 31989550 10.1007/978-1-0716-0223-2_4

[22] Schaarschmidt S, Fischer A, Zuther E et al. Evaluation of seven different RNA-seq alignment tools based on experimental data from the model plant Arabidopsis thaliana. Int J Mol Sci. 2020;21:1720. 10.3390/ijms21051720. 32138290 PMC7084517

[23] Vo K, Sharma Y, Paul A et al. Importance of transcript variants in transcriptome analyses. Cells. 2024;13:1502. 10.3390/cells13171502. 39273072 PMC11394320

[24] Kim P, Tan H, Liu J et al. FusionGDB 2.0: fusion gene annotation updates aided by deep learning. Nucleic Acids Res. 2022;50:D1221–30. 10.1093/nar/gkab1056. 34755868 PMC8728198

[25] Li H . Minimap2: pairwise alignment for nucleotide sequences. Bioinformatics. 2018;34:3094–100. 10.1093/bioinformatics/bty191. 29750242 PMC6137996

[26] Bolotin DA, Poslavsky S, Mitrophanov I et al. MiXCR: software for comprehensive adaptive immunity profiling. Nat Methods. 2015;12:380–1. 10.1038/nmeth.3364. 25924071

[27] Zhao X, Ren Z, Qi J et al. Gene fusion detection in long-read transcriptome sequencing data with GFvoter. Bmc Genomics [Electronic Resource]. 2025;26:670. 10.1186/s12864-025-11866-6. 40676509 PMC12269178

[28] Chang L, Zhou G, Soufan O et al. miRNet 2.0: network-based visual analytics for miRNA functional analysis and systems biology. Nucleic Acids Res. 2020;48:W244–51. 10.1093/nar/gkaa467. 32484539 PMC7319552

[29] Morris EK, Caruso T, Buscot F et al. Choosing and using diversity indices: insights for ecological applications from the German Biodiversity Exploratories. Ecol Evol. 2014;4:3514–24. 10.1002/ece3.1155. 25478144 PMC4224527

[30] Kogenaru S, Qing Y, Guo Y et al. RNA-seq and microarray complement each other in transcriptome profiling. Bmc Genomics [Electronic Resource]. 2012;13:629. 10.1186/1471-2164-13-629. 23153100 PMC3534599

[31] Nazarov PV, Muller A, Kaoma T et al. RNA sequencing and transcriptome arrays analyses show opposing results for alternative splicing in patient derived samples. Bmc Genomics [Electronic Resource]. 2017;18:443. 10.1186/s12864-017-3819-y. 28587590 PMC5461714

[32] Gao X, Yourick MR, Campasino K et al. An updated comparison of microarray and RNA-seq for concentration response transcriptomic study: case studies with two cannabinoids, cannabichromene and cannabinol. Bmc Genomics [Electronic Resource]. 2025;26:392. 10.1186/s12864-025-11548-3. 40264021 PMC12016467

[33] Carbonell-Sala S, Perteghella T, Lagarde J et al. CapTrap-seq: a platform-agnostic and quantitative approach for high-fidelity full-length RNA sequencing. Nat Commun. 2024;15:5278. 10.1038/s41467-024-49523-3. 38937428 PMC11211341

[34] Legnini I, Alles J, Karaiskos N et al. FLAM-seq: full-length mRNA sequencing reveals principles of poly(A) tail length control. Nat Methods. 2019;16:879–86. 10.1038/s41592-019-0503-y. 31384046

[35] Li Q, Keskus AG, Wagner J et al. Unraveling the hidden complexity of cancer through long-read sequencing. Genome Res. 2025;35:599–620. 10.1101/gr.280041.124. 40113261 PMC12047254

[36] Benesova S, Kubista M, Valihrach L. Small RNA-sequencing: approaches and considerations for miRNA analysis. Diagnostics (Basel). 2021;11:964. 10.3390/diagnostics11060964. 34071824 PMC8229417

[37] Song L, Ouyang Z, Cohen D et al. Comprehensive characterizations of immune receptor repertoire in tumors and cancer immunotherapy studies. Cancer Immunol Res. 2022;10:788–99. 10.1158/2326-6066.CIR-21-0965. 35605261 PMC9299271

[38] Seo K, Choi JK. Comprehensive analysis of TCR and BCR repertoires: insights into methodologies, challenges, and applications. Genomics Inform. 2025;23:6. 10.1186/s44342-024-00034-z. 39994831 PMC11853700

[39] Iwabuchi S, Tsukahara T, Okayama T et al. B cell receptor repertoire analysis from autopsy samples of COVID-19 patients. Front Immunol. 2023;14:1034978. 10.3389/fimmu.2023.1034978. 36911681 PMC9996338

[40] Jacobsen SB, Tfelt-Hansen J, Smerup MH et al. Comparison of whole transcriptome sequencing of fresh, frozen, and formalin-fixed, paraffin-embedded cardiac tissue. PLoS One. 2023;18:e0283159. 10.1371/journal.pone.0283159. 36989279 PMC10058139

[41] Previdere C, Bonin S, Cuttaia C et al. Are pre-analytical factors fully considered in forensic FFPE molecular analyses? A systematic review reveals the need for standardised procedures. Int J Legal Med. 2025;139:1439–52. 10.1007/s00414-025-03480-8. 40172636 PMC12170738

