# Supplementary material for: Systematic evaluation of long- and short-read RNA-seq for human peripheral blood
Source: NAR Mol Med. 2026 Jan 20;3(1):ugag006. doi: 10.1093/narmme/ugag006 (PMC12862385; doi:10.1093/narmme/ugag006)
Supplement: ugag006_Supplemental_Files [file ugag006_supplemental_files.zip › Supplementary_Figures_legends_v3.pdf]

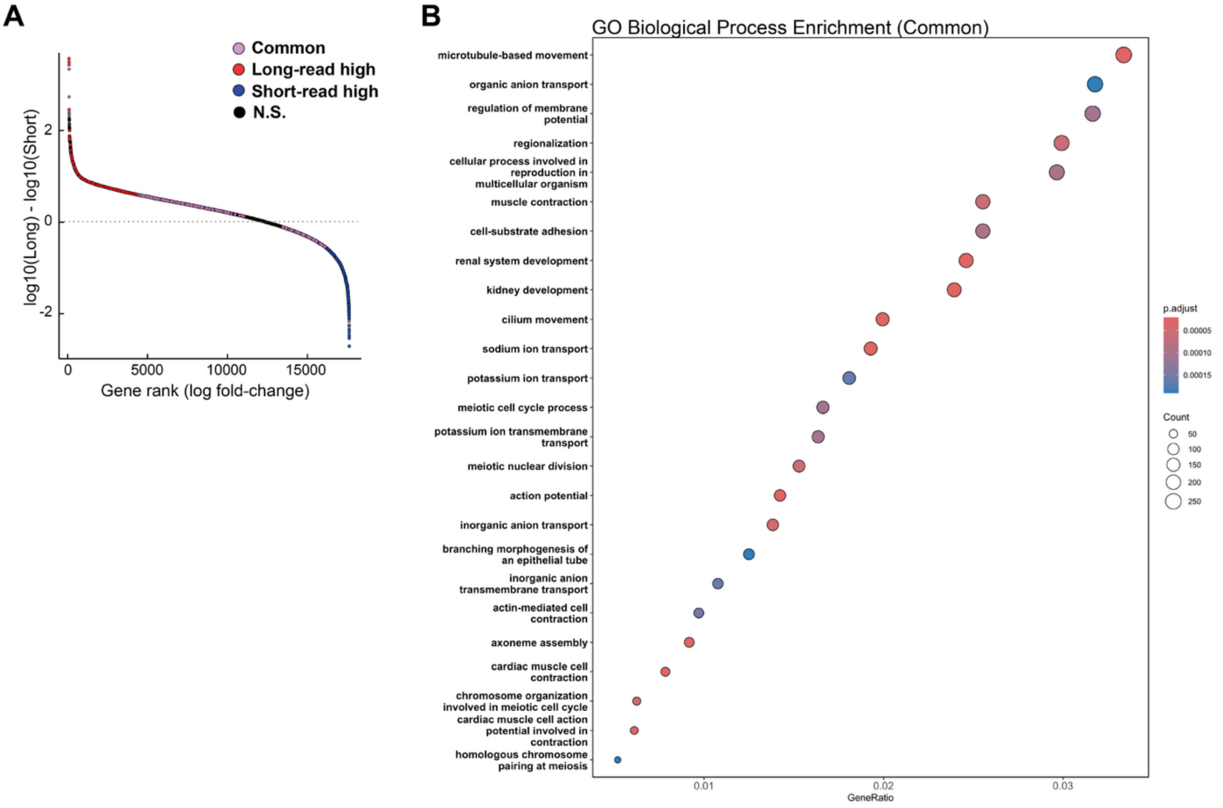

Supplementary Figure S1. Validation and functional analysis supporting transcriptome comparison. **A:** Scatter plot ranking genes by their log10 fold change [$\log_{10}$(Long) − $\log_{10}$(Short)]. Genes are sorted in descending order of differential expression. Red, blue, and violet indicate upregulation in long-read, short-read, or common in both, respectively. A gray dotted line at zero indicates no difference. **B:** GO Biological Process enrichment for genes classified as "common" based on DESeq2 paired differential expression analysis, in which no significant expression difference was detected between long-read and short-read RNA-seq (adjusted $p \geq 0.05$). These genes represent stable transcriptomic features consistently captured across platforms. Dot size reflects gene ratio and dot color indicates adjusted p-values.

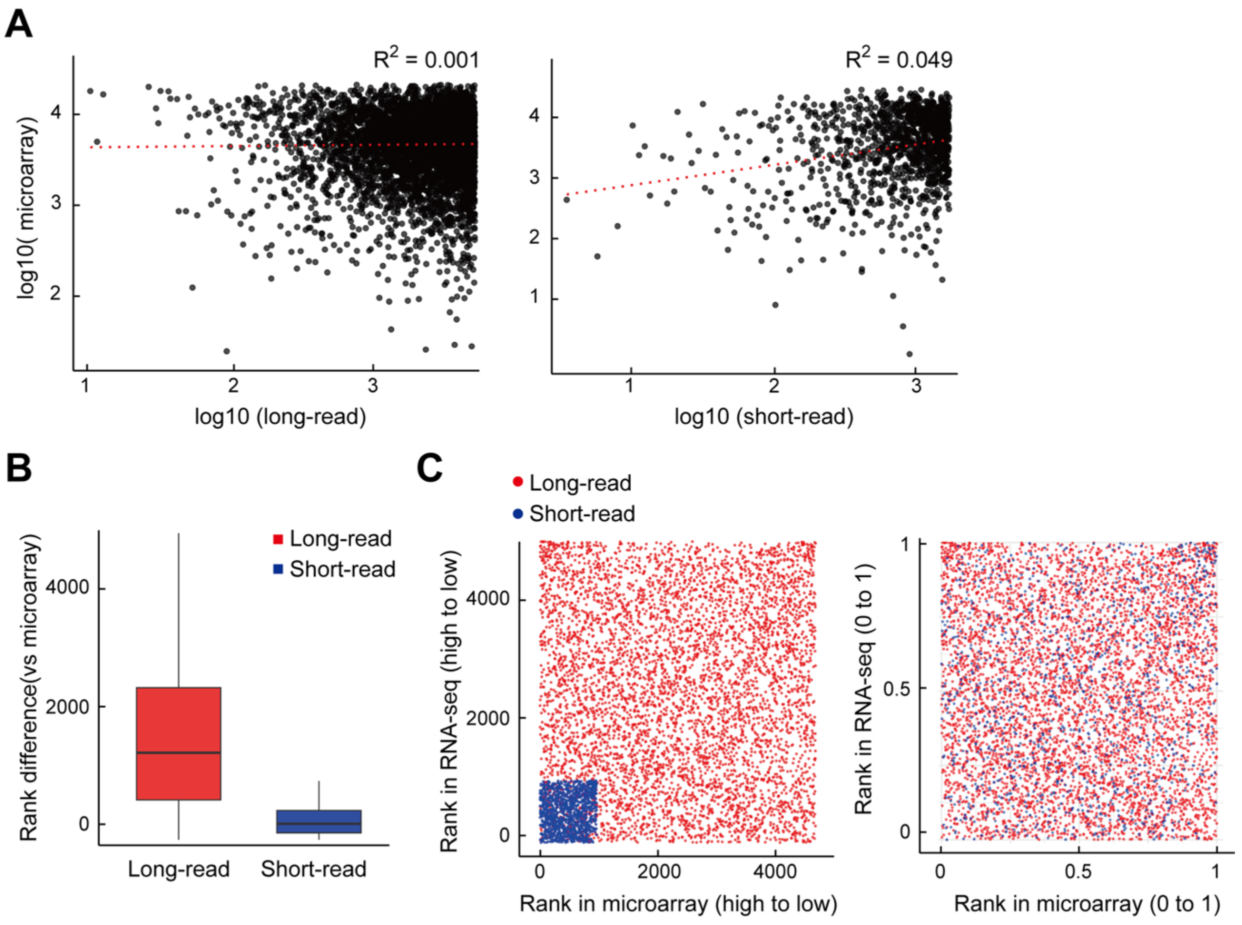

Supplementary Figure S2. Comparison of long-read, short-read, and microarray expression data. This figure summarizes comparative analyses between RNA-seq (long-read and short-read) and microarray data using a subset of genes significantly differentially expressed between long-read and short-read RNA-seq. Specifically, genes with TPM fold change > 2.0 and adjusted *p*-value < 0.05 in either direction (long > short: *n* = 5,539 genes; short > long: *n* = 1,139 genes) were selected, and only those also detected in the microarray dataset were included for downstream comparisons. **A:** Scatter plots comparing gene expression levels between RNA-seq and microarray platforms. The left panel shows log_{10}-transformed expression values of long-read RNA-seq (X-axis) versus microarray (Y-axis), and the right panel shows log_{10}-transformed short-read RNA-seq (X-axis) versus microarray (Y-axis). Each dot represents a gene detected in both datasets. Red dotted lines indicate the regression line, and the coefficient of determination (R^2) is annotated on each panel. **B:** Boxplots showing

the absolute rank differences between microarray and RNA-seq (long-read or short-read) for shared genes. The Y-axis indicates the absolute difference in gene expression rank compared to microarray data. Red and blue represent long-read and short-read RNA-seq, respectively. The central line within each box represents the median, while the box and whiskers indicate the interquartile range and overall spread of the rank differences. **C:** Rank correlation plots comparing the gene ranking (based on expression) between RNA-seq and microarray datasets. The X-axis represents gene rank in the microarray data (from highest to lowest), and the Y-axis shows the corresponding rank in long-read (red) or short-read (blue) RNA-seq. Each dot represents a gene. Genes from short-read RNA-seq are more concentrated in the lower rank region, while those from long-read RNA-seq are more widely dispersed, reflecting differences in concordance with the microarray platform. **D:** Scaled rank correlation plot, in which rank values for both RNA-seq, and microarray were normalized between 0 and 1. This revealed comparable variability between long-read and short-read RNA-seq when the number of paired genes was adjusted.

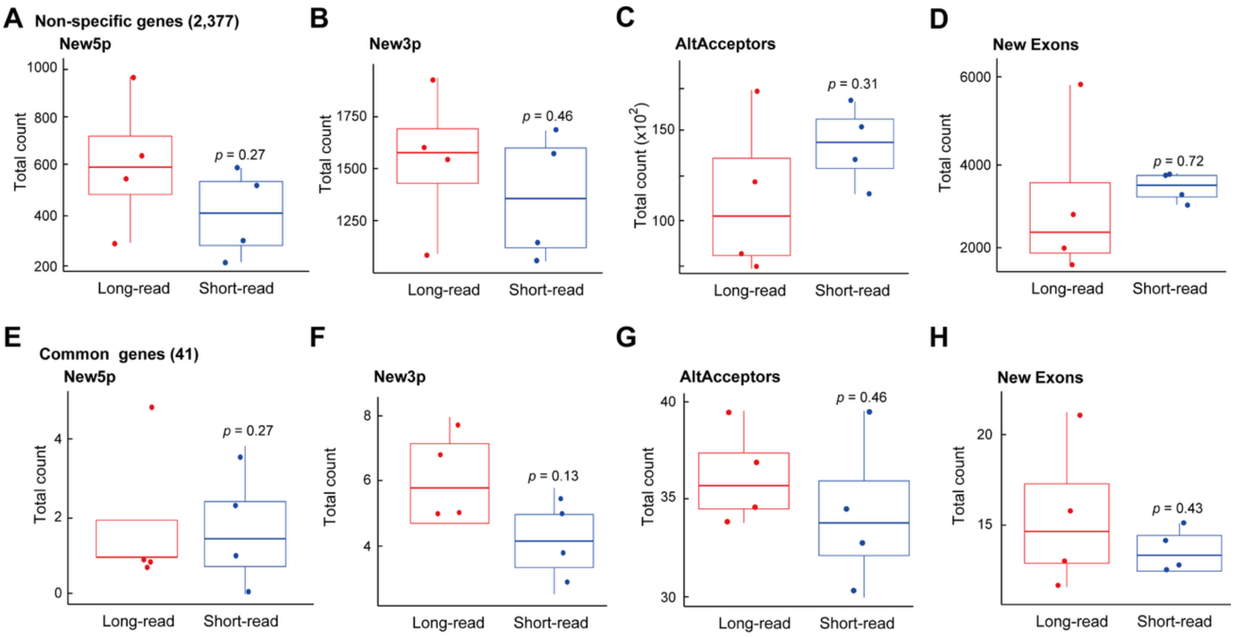

Supplementary Figure S3. Comparison of novel transcript elements between short-read and long-read RNA-seq within non-specific and common gene sets. **A-D**: Box plots showing the number of newly identified transcript features per gene in the non-specific group (n = 2,377 genes), defined as those detected in at least one sample from both short-read and long-read RNA-seq datasets. **A**: Number of novel 5′ ends (New5p), **B**: Number of novel 3′ ends (New3p). **C**: Number of alternative acceptor sites (AltAcceptors), **D**: Number of novel internal exons (NewExons). No statistically significant differences were observed between platforms in any feature category. **E–H**: Box plots showing the number of novel transcript features per gene in the common group (n = 41 genes), defined as those consistently detected across all eight datasets (i.e., in all four individuals by both platforms). **E**: Number of novel 5′ ends (New5p), **F**: Number of novel 3′ ends (New3p), **G**: Number of alternative acceptor sites (AltAcceptors), **H**: Number of novel internal exons (NewExons). No statistically significant differences were observed between short-read and long-read RNA-seq for any of the features.

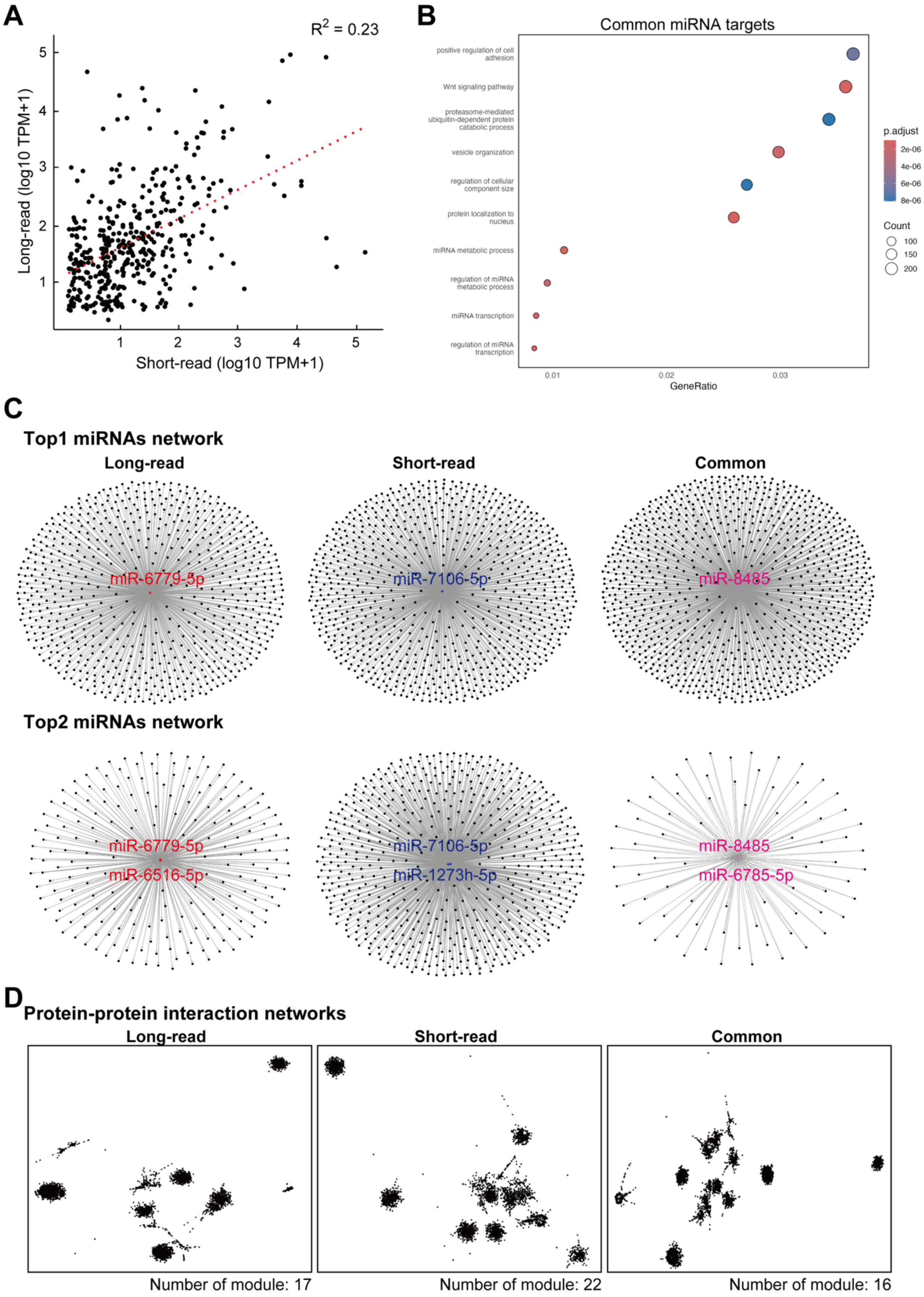
A
R^2 = 0.23
Long-read (log10 TPM+1)
Short-read (log10 TPM+1)
B
Common miRNA targets
positive regulation of cell adhesion
Wnt signaling pathway
proteasome-mediated ubiquitin-dependent protein catabolic process
vesicle organization
regulation of cellular component size
protein localization to nucleus
miRNA metabolic process
regulation of miRNA metabolic process
miRNA transcription
regulation of miRNA transcription
GeneRatio
p.adjust
Count
C
Top1 miRNAs network
Long-read
Short-read
Common
miR-6779-5p
miR-7106-5p
miR-8485
Top2 miRNAs network
miR-6779-5p
miR-6516-5p
miR-7106-5p
miR-1273h-5p
miR-8485
miR-6785-5p
D
Protein-protein interaction networks
Long-read
Short-read
Common
Number of module: 17
Number of module: 22
Number of module: 16

Supplementary Figure S4. Comparison of miRNAs between short-read and long-read RNA-seq. **A:** Scatter plot showing the correlation of miRNA expression levels ($\log_{10}$ TPM + 1) detected by both Long-read and Short-read platforms. Only miRNAs commonly detected by both platforms (n = 399) were included. A significant positive correlation was observed (Pearson r = 0.85, $p < 0.001$), indicating consistency in expression measurements for shared miRNAs. **B:** GO enrichment of targets of the 102 commonly detected miRNAs across both platforms. **C:** The miRNA-target gene networks corresponding to the top 1 and top 2 miRNAs for each platform, displayed in order: long-read, short-read, common. Node labels are shown to illustrate detailed gene-miRNA interactions. **D:** Modular structure of protein-protein interaction (PPI) networks constructed from miRNA target genes. The PPI networks derived from long-read-specific (left), short-read-specific (middle), and commonly detected (right) miRNA target gene sets are shown. Each dot represents a protein (node), and gray lines represent known protein-protein interactions. Nodes are grouped based on modularity clustering (Louvain algorithm), forming distinct functional modules. Although the number of modules varied slightly between platforms, the overall network topology appeared preserved across datasets. Number of module (cluster) is shown.

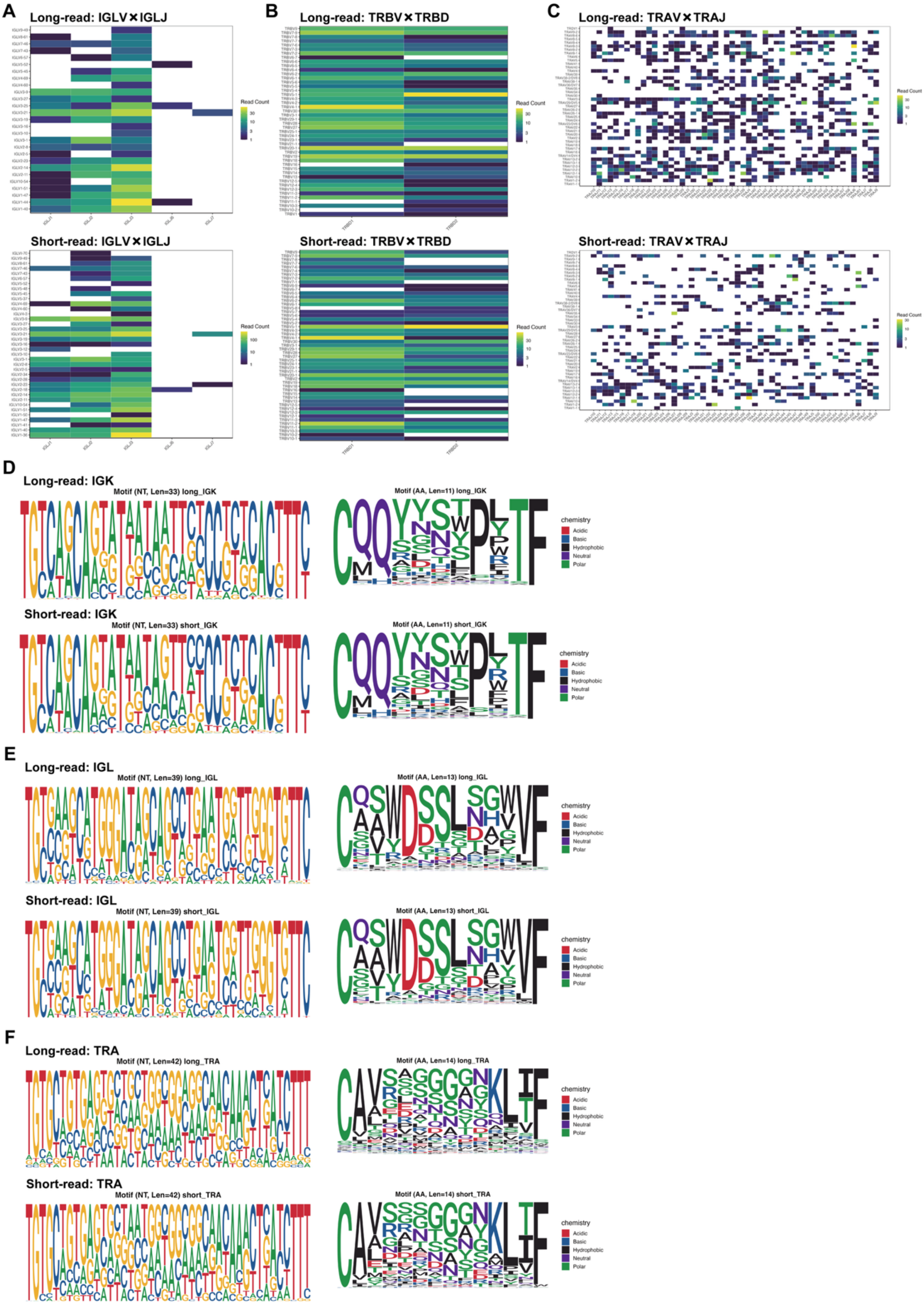
A
Long-read: IGLV×IGLJ
Short-read: IGLV×IGLJ
Read Count
B
Long-read: TRBV×TRBD
Short-read: TRBV×TRBD
C
Long-read: TRAV×TRAJ
Short-read: TRAV×TRAJ
D
Long-read: IGK
Motif (NT, Len=33) long_IGK
Motif (AA, Len=11) long_IGK
Short-read: IGK
Motif (NT, Len=33) short_IGK
Motif (AA, Len=11) short_IGK
chemistry
Acidic
Basic
Hydrophobic
Neutral
Polar
E
Long-read: IGL
Motif (NT, Len=39) long_IGL
Motif (AA, Len=13) long_IGL
Short-read: IGL
Motif (NT, Len=39) short_IGL
Motif (AA, Len=13) short_IGL
F
Long-read: TRA
Motif (NT, Len=42) long_TRA
Motif (AA, Len=14) long_TRA
Short-read: TRA
Motif (NT, Len=42) short_TRA
Motif (AA, Len=14) short_TRA

Supplementary Figure S5. Comparison of BCR and TCR repertoires between long-read and short-read sequencing using MiXCR. **A:** Heatmap of IGLV versus IGLJ gene combinations. Upper panel: long-read; Lower panel: short-read. Colors indicate read counts from yellow (high) through green to dark blue (low). **B:** Heatmap of TRBV versus TRBD gene combinations, with the same format as in A. **C:** Heatmap of TRAV versus TRAJ gene combinations. **D:** CDR3 motif logos of IGK chains. Left: nucleotide sequences; Right: amino acid sequences. Upper panels: long-read; Lower panels: short-read. In the amino acid logos, residues are color-coded by chemical properties: acidic (red), basic (blue), hydrophobic (black), neutral and polar (green). **E:** CDR3 motif logos of IGL chains, shown in the same format as in **D**. **F:** CDR3 motif logos of TRA chains. Diversity indices in A,E; Long-read: Shannon = 5.780, Simpson = 0.995, Short-read: Shannon = 6.022, Simpson = 0.994. B: Long-read: Shannon = 6.640, Simpson = 0.997. Short-read: Shannon = 6.252, Simpson = 0.9973. C&F: Long-read: Shannon = 6.922, Simpson = 0.998, Short-read: Shannon = 6.261, Simpson = 0.996. D: Long-read: Shannon = 6.125, Simpson = 0.996, Short-read: Shannon = 6.566, Simpson = 0.996.
