# Supplementary material for: Systematic evaluation of long- and short-read RNA-seq for human peripheral blood
Source: NAR Mol Med. 2026 Jan 20;3(1):ugag006. doi: 10.1093/narmme/ugag006 (PMC12862385; doi:10.1093/narmme/ugag006)
Supplement: ugag006_Supplemental_Files [file ugag006_supplemental_files.zip › Supplementary_Table_1.docx]

**Supplementary Table 1.**

Summary of short- and long-read RNA sequencing statistics mapped to the T2T reference genome

**Short-read**

| ID | Total reads | Longest read | Read mapped | Total genes | Total exons | Exon-exon junctions | Intergenic |
| --- | --- | --- | --- | --- | --- | --- | --- |
| HD1 | 137,028,187 | 76 | 97.35% | 96.61% | 88.13% | 23.33% | 3.39% |
| HD2 | 160,496,176 | 76 | 96.68% | 94.59% | 85.06% | 22.20% | 5.41% |
| HD3 | 28,043,629 | 76 | 97.59% | 96.61% | 79.50% | 19.35% | 3.39% |
| HD4 | 25,929,399 | 76 | 96.93% | 96.65% | 82.22% | 20.16% | 3.35% |

Note: The short-read sequencing for HD1 and HD2 was composed of six sub-read fastq files, with an average of 27,405,637 (HD1) and 26,749,529 (HD2) total reads per fastq file.

**Long-read**

| ID | Total read | Longest read | Read mapped | Total Gene | Total Exon | Exon-exon | Intergenic |
| --- | --- | --- | --- | --- | --- | --- | --- |
| HD1 | 7,623,163 | 315,571 | 89.31% | 97.03% | 79.87% | 73.67% | 2.97% |
| HD2 | 7,499,817 | 343,228 | 89.47% | 97.11% | 83.08% | 76.57% | 2.89% |
| HD3 | 7,093,631 | 313,194 | 90.16% | 96.54% | 72.32% | 66.51% | 3.46% |
| HD4 | 7,628,890 | 380,895 | 88.51% | 96.54% | 74.86% | 69.18% | 3.46% |
