# Supplementary material for: Systematic evaluation of long- and short-read RNA-seq for human peripheral blood
Source: NAR Mol Med. 2026 Jan 20;3(1):ugag006. doi: 10.1093/narmme/ugag006 (PMC12862385; doi:10.1093/narmme/ugag006)
Supplement: ugag006_Supplemental_Files [file ugag006_supplemental_files.zip › Supplementary_Table_2.docx]

**Supplementary Table 2.**

Diversity indices (Shannon and Simpson) for each immunoglobulin and T cell receptor chain across long-read (top) and short-read sequencing (bottom).

| Long-read | Shannon | Simpson |
| --- | --- | --- |
| IGH | 8.592 | 0.999 |
| IGK | 6.125 | 0.996 |
| IGL | 5.780 | 0.995 |
| TRA | 6.922 | 0.998 |
| TRB | 6.640 | 0.997 |
| TRD | 3.891 | 0.977 |
| TRG | 4.728 | 0.968 |

| Short-read | Shannon | Simpson |
| --- | --- | --- |
| IGH | 5.888 | 0.994 |
| IGK | 6.566 | 0.996 |
| IGL | 6.022 | 0.994 |
| TRA | 6.261 | 0.996 |
| TRB | 6.252 | 0.9973 |
| TRD | 1.629 | 0.742 |
| TRG | 2.751 | 0.859 |
